# Tetrahydropalmatine may alleviate doxorubicin-induced renal injury by activating the Sirt3-mediated Nrf2/HO-1 pathway

**DOI:** 10.1186/s13062-026-00773-9

**Published:** 2026-04-30

**Authors:** Wei Wang, Yuanyuan Hu, Yan Xu, Ning Ding, Jiping Wei, Cairong Li, Yong Chen

**Affiliations:** 1 School of Pharmacy, Xianning Medical College, Hubei University of Science and Technology, Xianning, 437100 China; 2 School of Basic Medical Sciences, Xianning Medical College, Hubei University of Science and Technology, Xianning, 437100 China; 3 Second Affiliated Hospital, Clinical Medical School, Xianning Medical College, Hubei University of Science and Technology, Xianning, 437100 China; 4 Department of Nephrology, Xianning Central Hospital, The First Affiliate Hospital of Hubei University of Science and Technology, Xianning, 437100 China

**Keywords:** Tetrahydropalmatine, Doxorubicin, Podocytes, Renal injury, Sirt3, Nrf2/HO-1 pathway

## Abstract

**Background:**

Doxorubicin (DOX) is a widely used broad-spectrum chemotherapy drug, but its severe organ toxic and side effects limit its clinical application. Tetrahydropalmatine (THP) protects against DOX-induced renal injury, yet its underlying mechanism remains unclear. This study aimed to verify THP’s protective effect and explore if it acts via the SIRT3-mediated Nrf2/HO-1 signaling pathway.

**Methods:**

This study adopted an integrated in vivo and in vitro research system to explore the relevant mechanisms. In vivo, a DOX-induced mouse renal injury model was established with THP intervention; renal function was evaluated by detecting serum creatinine (Scr) and blood urea nitrogen (BUN), while renal injury severity was assessed via urine microalbumin (mALB) and urine albumin/creatinine ratio (UACR). Renal histopathological changes including glomerular injury and tubulointerstitial fibrosis were observed, and subcellular structures such as podocyte integrity and mitochondrial morphology were analyzed by transmission electron microscopy (TEM); additionally, the expression of pathway-related proteins was detected. In vitro, a DOX-induced cell injury model was constructed using mouse podocytes (MPC-5), and Western blot and immunofluorescence techniques were employed to determine the expression of key molecules in the Sirt3-Nrf2/HO-1 pathway, combined with in vivo and in vitro experiments to clarify the underlying mechanism.

**Results:**

THP significantly attenuated DOX-induced renal injury, improved renal function (reduced Scr/BUN), mitigated histopathological/mitochondrial abnormalities, and preserved podocyte integrity. Molecularly, THP upregulates podocyte markers Nephrin/Podocin, enhances antioxidant capacity (increased GSH, SOD, and CAT, decreased MDA), inhibits inflammation (downregulated IL-6, IL-1β, and TNF-α), and suppresses apoptosis (downregulated Bax/caspase-3, upregulated Bcl-2). Notably, both in vivo and in vitro data link THP’s protection to SIRT3-mediated Nrf2/HO-1 pathway activation.

**Conclusion:**

THP protects against DOX-induced renal injury in mice. Mechanistically, this may involve activating the SIRT3-mediated Nrf2/HO-1 pathway, improving podocyte function, and inhibiting oxidative stress, inflammation, and apoptosis.

**Supplementary Information:**

The online version contains supplementary material available at 10.1186/s13062-026-00773-9.

## Introduction

Doxorubicin (DOX), first isolated from Streptomyces species, belongs to the anthracycline class of antibiotics and is widely used for treating various types of cancer [1]. However, its clinical application is significantly limited due to severe nephrotoxicity [2]. Renal damage from DOX is insidious and progressive, leading to acute kidney injury or chronic kidney disease, which greatly reduces the quality of patient survival.

The mechanism of nephrotoxicity of DOX involves multi-pathway interactions. On the one hand, DOX accumulates in renal tubular epithelial cells, disrupting mitochondrial function and inducing DNA damage [3]; on the other hand DOX activates inflammatory pathways, such as NF-κB, and releases pro-inflammatory factors, exacerbating inflammatory responses in the kidney [4]. Oxidative stress and inflammatory responses are major contributors to the progression of kidney diseases, including diabetic nephropathy, chronic kidney disease, and acute kidney injury. Under normal physiological conditions, a dynamic balance exists between oxidants and antioxidants. However, when this equilibrium is disrupted in favor of oxidation, oxidative stress occurs, resulting in excessive production of reactive oxygen species (ROS) [5]. An overabundance of ROS can induce both structural and functional damage to renal proteins and nucleic acids, ultimately leading to cellular injury, microvascular dysfunction, and glomerular impairment [6]. Similarly, inflammation is central to the pathophysiology of renal diseases. Inflammation in the kidneys triggers the activation of immune cells and facilitates the liberation of pro-inflammatory cytokines like IL-6, IL-1β, and TNF-α [7]. The accumulation of these inflammatory mediators in the kidney contributes to tubular injury and fibrosis, reducing the glomerular filtration rate (GFR) and exacerbating glomerular damage [8]. Additionally, research has indicated that elevated oxidative stress and inflammation can further promote apoptosis [9]. Although doxorubicin-induced kidney injury and secondary fibrosis have become urgent clinical challenges in the management of drug-induced nephrotoxicity, current clinical interventions remain limited in efficacy and are associated with significant adverse effects [10]. Against this backdrop, natural medicines that are widely available and relatively safe have gradually emerged as an important research area for the management of chemotherapy-related kidney injury due to their potential renoprotective effects.

Podocin and Nephrin, as key transmembrane receptor proteins, are located at the junctions of differentiated podocytes, forming a specialized structure known as the slit diaphragm [11]. This structure is critical for the barrier function of the glomerular capillary wall, and its integrity directly determines the maintenance of normal filtration. Mutations in Podocin and Nephrin have been shown to impair the structure and function of the slit diaphragm, leading to podocyte damage and nephropathy [12]. In addition, exposure to exogenous factors such as DOX promotes renal fibrosis (via the TGF-β/Smad pathway) and podocyte apoptosis in mice, ultimately leading to glomerulosclerosis and interstitial fibrosis [13]. Further studies found that DOX, upon accumulation in podocytes, generates excess ROS by attacking the mitochondrial electron transport chain, triggering impaired energy metabolism and mitochondrial membrane potential collapse, thereby exacerbating apoptosis and accelerating podocyte detachment and death [14]. Notably, the importance of the maintenance of podocyte morphological integrity for glomerular function has been widely emphasised, e.g. Kopp et al. pointed out that it is a decisive factor in safeguarding normal filtration function [15].

Sirtuin 3 (Sirt3), a key enzyme of the sirtuin family, functions as an NAD^+^-dependent deacetylase within the mitochondria, where it modulates the acetylation of various substrates involved in energy metabolism [16]. Studies have demonstrated that Sirt3 exerts nephroprotective effects by reducing ROS production, inhibiting NLRP3 inflammasome activation, alleviating oxidative stress, and decreasing pro-inflammatory cytokine levels [17]. The nuclear factor erythroid 2-related factor 2/heme oxygenase-1 (Nrf2/HO-1) signaling pathway plays a critical role in regulating the renal antioxidant defense system and modulating the expression of anti-inflammatory genes [18]. Under pharmacological intervention, Nrf2 translocated to the nucleus, activated downstream HO-1, and suppressed intracellular inflammation and oxidative stress, thereby exerting renoprotective effects [19].

Accumulating reports have demonstrated that natural products exhibit promising pharmacological activities and therapeutic value in the treatment of kidney disease, providing important references for our exploration of natural product-based protective strategies against chemotherapy-induced kidney injury [20]. Tetrahydropalmatine (THP), an isoquinoline alkaloid, is widely found in various herbal remedies [21]. THP is primarily extracted from Stephania species and exhibits a broad spectrum of biological activities, including anti-inflammatory, antioxidant, anticoagulant, and antiviral properties [22]. Research has demonstrated that THP shows therapeutic potential in treating neurological, cardiovascular, hepatic, and pulmonary injuries, as well as diabetes mellitus [23–25]. Moreover, recent studies show that L-tetrahydropalmatine (L-THP), the levo-isomer of THP, selectively inhibits organic cation transporter 2 (OCT2) to reduce renal cisplatin accumulation, thereby mitigating cisplatin-induced nephrotoxicity in primary renal tubular cells without compromising cisplatin’s antitumor efficacy [26]. Given the multi-target pharmacological profile of THP (including anti-inflammatory and antioxidant activities), its renoprotective effects against cisplatin-induced kidney injury likely involve a synergistic interplay between OCT2 inhibition and these inherent bioactivities, thereby offering novel perspectives for developing comprehensive renoprotective strategies that combine targeted transporter regulation with multi-pathway intervention.

Notably, the organ-protective potential of THP has been progressively validated. Based on these insights, the present study establishes a DOX-induced nephropathy model via intravenous injection and investigates the mechanism by which THP alleviates DOX-induced renal injury and fibrosis through the Sirt3-Nrf2/HO-1 signaling pathway, providing new experimental evidence for the targeted intervention of drug-induced nephrotoxicity and the prevention and treatment of CKD.

## Materials and methods

### Animal experiment

**Fig. 1:**
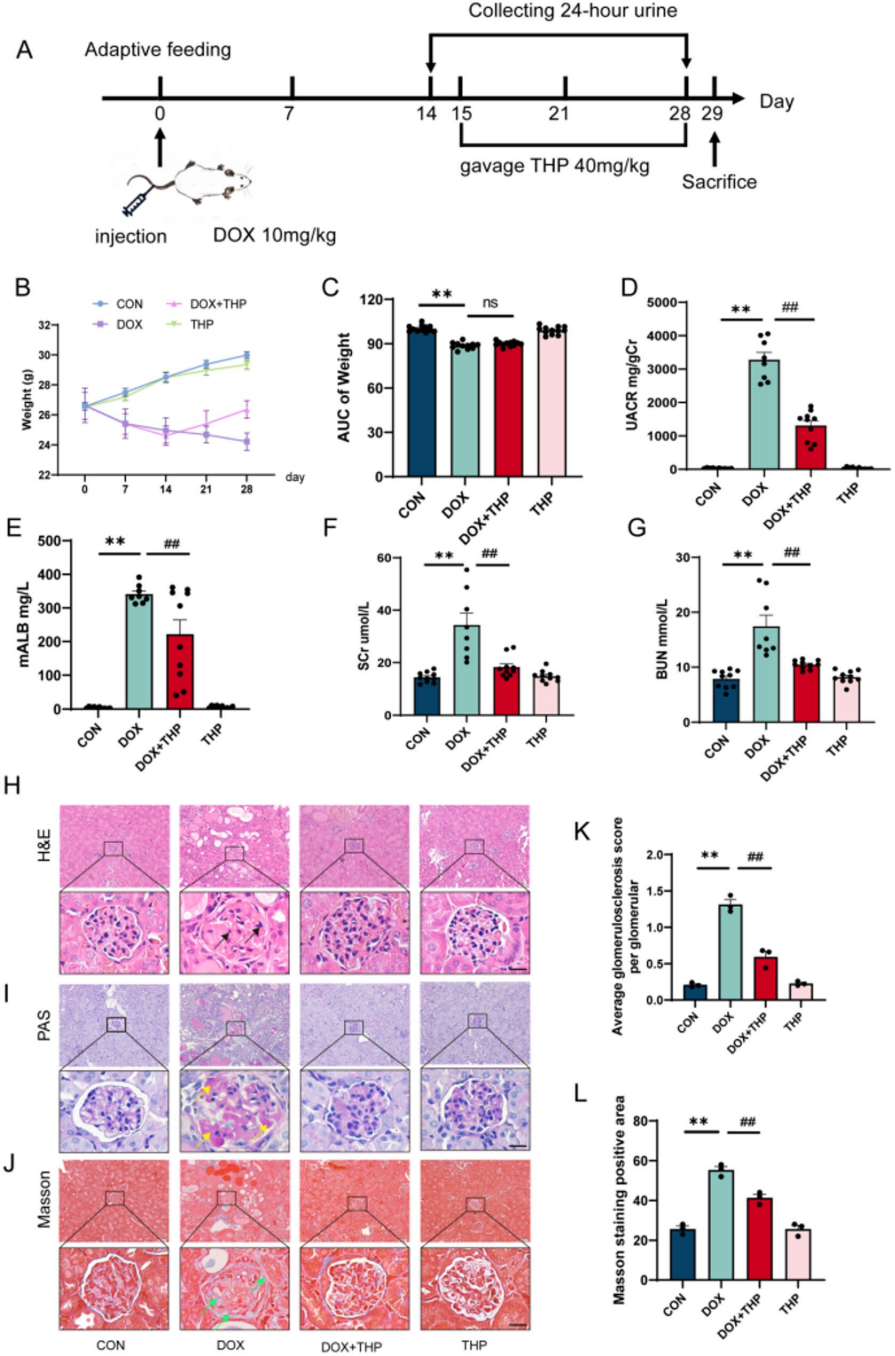
THP ameliorates DOX-induced renal dysfunction in mice. (**A**) Schematic diagram of the animal experimental design. (**B**) Changes in body weight in different groups of mice (*n* = 8–10). (**C**) Area under the curve of body weight. (**D**-**G**) Levels of urinary albumin-to-creatinine ratio (UACR), urinary albumin (mALB), serum creatinine (Scr), and blood urea nitrogen (BUN) in different groups (*n* = 8–10). Panels **H**-**J**: Upper: 100×, scale bar = 50 μm; Lower insets: 400×, scale bar = 20 μm. (**H**) H&E staining: Black arrows indicate glomerular swelling and inflammatory cell infiltration; (**I**) PAS staining: Yellow arrows indicate glycogen deposition and glomerular sclerosis; (**J**) Masson staining: Green arrows indicate interstitial fibrosis. (**K**) Average glomerulosclerosis score per glomerular (*n* = 3 per group, 6 glomeruli evaluated for each mouse). (**L**) Mean percentage of positively stained areas of Masson staining. Data are presented as mean ± SEM. **P < 0.01, vs. control group; ^##^*P* < 0.01, vs. DOX group; ns indicates no significance

Forty male BALB/c mice (22 ± 2 g, 6–8 weeks) were obtained from Hubei Bainter Biotechnology Co. (Approval No.: SCXK2021-0027, Wuhan, China). During the experiment, mice were housed under controlled conditions (20–26 °C, 40–70% humidity). Mice were maintained under a 12-hour light/dark cycle with ad libitum access to food and water. Each cage contained a maximum of five mice, and the bedding was replaced thrice a week. The mice were randomly allotted into four experimental groups, with each group consisting of 10 mice: control group (CON), model group (DOX), model + THP group (DOX + THP), and THP-treated group (THP). After an acclimatization period, a single injection of DOX (10 mg/kg) [27–29]was given via the tail vein to the mice in the DOX and DOX + THP groups. Conversely, an equivalent volume of saline was administered to the mice in the CON and THP groups. Starting on day 14, mice in the DOX + THP and THP groups were given daily oral doses of THP (40 mg/kg) [23, 30, 31] (only for dose range guidance), and the doses of all reagents used in this study were screened and determined via preliminary experiments; while those in the CON and DOX groups were administered a commensurate volume of solvent. On day 29, all mice were sacrificed. Throughout the study, body weight was monitored, and Twenty-four-hour urine samples were obtained according to the experimental protocol (Fig. 1A). Upon completion of the experiment, serum samples were obtained for biochemical analysis, while kidney tissues were collected for histopathological examination and further analyses. All experimental procedures involving animals were evaluated and authorized by the Animal Ethics Committee of Hubei University of Science and Technology (HBUST) and conducted by HBUST Animal Ethics Committee guidelines.

### Chemicals and reagents

Doxorubicin hydrochloride 97% (No: A183027, Aladdin, Shanghai, China) was dissolved in saline (No: L23101104, Kelun, Sichuan, China) and administered as a single tail vein injection at a dose of 10 mg/kg [27]. Tetrahydropalmatine 97% (T101543, Aladdin, Shanghai, China) was dissolved in DMSO (Sigma, USA) and diluted in 0.5% carboxymethyl cellulose solution to a final concentration of 40 mg/kg. It was administered via gavage once daily for two weeks. Podocin (No: DF8593), Nephrin (No: AF7951) and Cleaved-caspase3 (No: AF7022) were purchased from Affinity (Changzhou, China); β-Tubulin (No: AC021), IL-6 (No: A0286) were purchased from Abclonal (Wuhan, China); IL-1β (No: 41059-2), TNF-α (No: #41504), β-actin (No: 52901-2), HRP Goat Anti-Rabbit IgG Antibody (No: #L3012) and HRP Goat Anti-Mouse IgG Antibody (No: #L3012) were purchased from SAB (Maryland, USA); Bax (No: bs-0127R) and Bcl2 (No: bs-0032R) were purchased from Bioss (Beijing, China); Sirt3 (No: 5490 S) was purchased from Cell Signaling (Massachusetts, USA); NRF2 (No: 16396-1-AP) and HO-1 (No: 10701-1-AP) were purchased from Proteintech (Wuhan, China); We directly sourced the Cy3-labeled goat anti-mouse IgG (No: GB21301), GAPDH (No: GB15002), and the Alexa Fluor 488-labeled goat anti-rabbit IgG (No: GB25303) from Servicebio (Wuhan, China). See Table 1 for details of dilution ratios and applications.

**Table 1:** Antibodies used in this study

Primary antibodies	Host	Dilution and supplier	Catalog number	Application
Nephrin	Rabbit	1:100; Affinity	AF7951	IHC, IF
Nephrin	Rabbit	1:1000; Affinity	AF7951	WB
Podocin	Rabbit	1:1000; Affinity	DF8593	WB
β-actin	Rabbit	1:1000; SAB	52901-2	WB
β-tubulin	Mouse	1:5000; Abclonal	AC021	WB
IL-6	Rabbit	1:1000; Abclonal	A0286	WB
IL-1β	Rabbit	1:1000; SAB	41059-2	WB
TNF-α	Rabbit	1:1000; SAB	#41,504	WB
Bax	Rabbit	1:1000; Bioss	bs-0127R	WB
Bcl2	Rabbit	1:1000; Bioss	bs-0032R	WB
Cleaved-caspase3	Rabbit	1:1000; Affinity	AF7022	WB
Sirt3	Rabbit	1:1000; Cell Signaling	5490 S	WB
Sirt3	Rabbit	1:200; Cell Signaling	5490 S	IF
Nrf2	Rabbit	1:2000; Proteintech	16396-1-AP	WB
Nrf2	Rabbit	1:200; Proteintech	16396-1-AP	IF
HO-1	Rabbit	1:1000; Proteintech	10701-1-AP	WB
GAPDH	Mouse	1:8000; Servicebio	GB15002	WB

### Biochemical measurement

Mouse blood was left at room temperature for 2–3 h. Subsequently, serum was isolated by centrifugation at 3000 rpm for 15 min at 4 °C. The urine and serum samples were analyzed for the UACR, mALB, Scr, and BUN levels using an AU-680 fully automated biochemical analyzer (Beckman Coulter, USA).

### Measurement of MDA, GSH, SOD, and CAT

Briefly, tissue homogenates were prepared by adding approximately 20 mg of fresh kidney tissue to 300 µL of pre-cooled saline. The sample was subjected to centrifugation at 1,200 rpm for 15 min at 4 °C. After centrifugation, a total of 200 µL of the supernatant was carefully collected. Reagents for testing MDA (No: A003-1-2), GSH (No: A006-2-1), T-SOD (No: A001-3-2), and CAT (No: A007-1-1) were added in the correct amounts following the instructions in the provided kits(Jiancheng Bioengineering Institute, Nanjing). After the reaction, an appropriate volume of supernatant was transferred to a 96-well plate, and absorbance was measured using a microplate reader (BioTek, Vermont, USA).

### Histopathology

The kidneys of the mice were placed in test tubes containing 4% paraformaldehyde (PFA) and fixed overnight at 4 °C. Once fixed, the tissues underwent paraffin embedding, sectioning, and were readied for histological examination [32]. H&E, PAS, and Masson staining procedures were performed in accordance with the manufacturer’s instructions by using the corresponding kits from Solarbio, Beijing, China. A light microscope served as the tool to capture the histological images (Olympus, Tokyo, Japan). Quantification was performed by measuring the percentage of positively stained glomerular areas with ImageJ software (National Institutes of Health, USA).

### Immunohistochemistry

For immunohistochemical (IHC) analysis, we referenced previous research [33]. Kidney tissue samples were fixed in 4% paraformaldehyde for 48 h, embedded in paraffin, and sectioned into 8-µm-thick slices. Sections were mounted onto slides, fixed at room temperature for 1 h, then washed thrice with PBS. They were incubated in 2 M HCl at 37 °C for 1 h, neutralized with boric acid (pH 8.5) for 10 min, and blocked with 10% normal goat serum for 1 h to prevent non-specific binding. Next, sections were incubated overnight in a humid environment with primary antibodies (specific antibodies in Table 1). After three PBST washes, sections were incubated with horseradish peroxidase-labeled secondary antibody. Diaminobenzidine substrate visualized staining, and images were captured via a BX51 microscope. Slides were counterstained with hematoxylin; protein expression was assessed by IHC, with positive pixels of brown IHC staining and their intensity quantified using ImageJ (USA) software.

### Immunofluorescence

At 24 h post renal tissue sectioning or cell culture, samples were fixed in 4% buffered paraformaldehyde for 15 min, then washed three times with PBS (5 min each). After permeabilization with 0.5% Triton X-100 (room temperature, 5 min), samples were washed three times with PBS and blocked with goat serum for 30 min. Post-blocking, samples were incubated overnight at 4 °C with primary antibodies against Sirt3, Nrf2, and HO-1. The next day, slides or dishes were washed three times with PBS and incubated with secondary antibodies in the dark for 1 h. Finally, samples were incubated with DAPI for 5 min for nuclear staining and mounted with anti-fluorescence quencher.

### Western blot

Proteins were extracted and their concentrations determined. Samples were boiled and separated by 10% SDS-PAGE. After electrophoresis, proteins were transferred to PVDF membranes. To block non-specific binding, membranes were incubated with 5% skimmed milk in TBST for 60 min. After blocking, the membranes were incubated overnight at 4 °C with primary antibodies against the key target proteins including Sirt3, Nrf2, HO-1, Bax, and Bcl-2. Detailed information for all primary antibodies, including the target protein, manufacturer, catalog number, and dilution ratio, is provided in Table 1. The next day, membranes were washed three times with TBST (5 min each), then incubated with secondary antibody for 1 h. Protein expression was detected via chemiluminescence and imaged using the ChemiDoc™ Touch Imaging System (Bio-Rad, CA, USA).

### Cell culture

Mouse Podocyte Clone-5 (MPC-5) were cultured in 1640 medium (No: 8123267, Gibco), a gift from the Institute of Pharmaceutical Research, HBUST, Faculty of Medicine, Hubei Province, China. We enriched the 1640 medium by adding 10% fetal bovine serum and 1% penicillin-streptomycin, and then maintained the MPC-5 cells in an incubator at 37 °C with 5% CO2. DOX (0.5 µM), THP (80 µM), and 3-TYP (0.5 µM) were added via medium change and incubated continuously for 24 h.

### CCK-8 cell viability assay

Concentrations were optimized based on preliminary CCK-8 assays (Fig. 5A-C) and literature. MPC-5 cells were trypsinized, seeded into a 96-well plate at a density of 6,000 cells per well, and incubated for 24 h to allow attachment. Afterward, the culture medium was replaced with fresh medium containing the test compounds, and the cells were incubated for an additional 24 h. Next, each well had 10 µL of the CCK − 8 reagent (Kerui, Wuhan) added to it, and the plate was then incubated for 1 h at 37 °C. We measured the absorbance (OD value) at a 450-nm wavelength with the use of a microplate reader, the BioTek Synergy H1. Wells containing only the medium were used as the blank control. We determined the cell viability by contrasting the OD values of the treated groups with those of the untreated control group and then presented it as a percentage.

### Reverse transcription and real-time quantitative PCR

Total RNA was extracted from the samples via the Trizol method. Its concentration and purity were determined by measuring the A260/A280 ratio, which was between 1.8 and 2.0. We conducted reverse transcription on the RNA by using the Reverse Transcription Kit (017E2260DA, Vazyme, Nanjing, China). Then, qPCR was executed with ChamQ SYBR qPCR Master Mix (027E2232EA, Vazyme, Nanjing, China). The amplification program was as follows: an initial denaturation at 95 °C for 30 s, and then 40 cycles of 95 °C for 5 s and 60 °C for 30 s. The primer sequences are presented in Table 2, and the relative expression levels of the target genes were determined through the application of the 2^−ΔΔCt^ method.

**Table 2:** Primer Sequence for qPCR

Gene	Sequence (5’->3’)	Length
IL-6	Forward: GTCCTTCCTACCCCAATTTCCA	79
	Reverse: TGGTCTTGGTCCTTAGCCAC	
IL-1β	Forward: ATGGAAGTCTGTCTGCTCAGTA	145
	Reverse: GACAGCCCAGGTCAAAGGTT	
THF-α	Forward: GTGACAAGCCTGTAGCCCAC	182
	Reverse: GCAGCCTTGTCCCTTGAAGA	
GAPDH	Forward: GTCAAGGCCGAGAATGGGAA	237
	Reverse: CTCGTGGTTCACACCCATCA	
Sirt3	Forward: GGGAGTGTTACAGGTGGGAG	207
	Reverse: GCTCCCTGGGGATCTGAAGT	
Sirt2	Forward: TTCTTGTTTCCGCTGCCGTC	100
	Reverse: AGATGACCTTGCGGCGGTC	
Sirt1	Forward: TCGGCTACCGAGGTCCATA	136
	Reverse: ACAATCTGCCACAGCGTCAT	

### Transmission electron microscopy (TEM)

Tissue samples were preserved in 2.5% glutaraldehyde at 4 °C for 24 h, followed by a 2-hour post-fixation in 1% osmium tetroxide. After sequential dehydration with ethanol (50% to 100%), the samples were embedded in Epon 812 resin and cured at 60 °C for 48 h. Ultrathin Sects.  (70–90 nm thick) were prepared using a Leica Ultracut microtome, mounted on copper grids, and post-stained with uranyl acetate for 15 min followed by lead citrate for 5 min. Micrographs were captured using a Gatan Orius SC1000 digital camera. For quantitative analysis, three independent biological replicates were examined, and representative images were selected based on structural clarity and artifact-free fields.

### Statistical analysis

Experimental data are presented as mean ± standard error of the mean (SEM). All statistical analyses were performed using GraphPad Prism 9.0 software (GraphPad Software, USA). Unless otherwise stated, experiments were replicated at least three times under independent conditions. For comparisons involving multiple groups, statistical significance was assessed by one-way ANOVA followed by Tukey’s post hoc test. *P* < 0.05 was applied to determine statistical significance.

## Results

### THP ameliorated DOX-induced renal dysfunction and pathological features in mice

Initially, as shown in Fig. 1B–C, mice injected with DOX exhibited a progressive decline in body weight compared with normal controls. After four weeks, the area under the curve (AUC) of body weight in DOX group was significantly lower than that in control group (*P* < 0.01). Although THP treatment slightly increased body weight, this change was not statistically significant (*P* > 0.05). Additionally, Fig. 1D-F demonstrated that DOX-treated mice exhibited significant increases in the UACR, mALB, Scr, and BUN (*P* < 0.01). Following THP treatment, these parameters significantly decreased. It is well known that renal function indicators are crucial in the assessment of kidney disease. Our findings suggested that THP exerted renoprotective effects in DOX-induced renal injury.

To further assess the renoprotective effects of THP on DOX-induced kidney damage, histological analysis of kidney sections was performed using H&E, PAS, and Masson’s trichrome staining. H&E staining (Fig. 1H) revealed that DOX-treated mice exhibited tubular dilation, glomerular swelling, and extensive inflammatory infiltration compared with controls. PAS staining (Fig. 1I) demonstrated glomerulosclerosis in the DOX group, characterized by glomerular hypertrophy, adhesion, and basement membrane thickening. Masson’s trichrome staining (Fig. 1J) further confirmed fibrosis in and around the glomeruli, with substantial interstitial collagen deposition. In contrast, THP treatment significantly improved glomerular and tubular morphology, reduced collagen deposition and inflammatory infiltration, and alleviated both glomerulosclerosis and fibrosis. Above experimental results confirmed that THP effectively mitigated DOX-induced renal injury.

### THP attenuated DOX-induced podocyte and mitochondria damage in mice

**Fig. 2:**
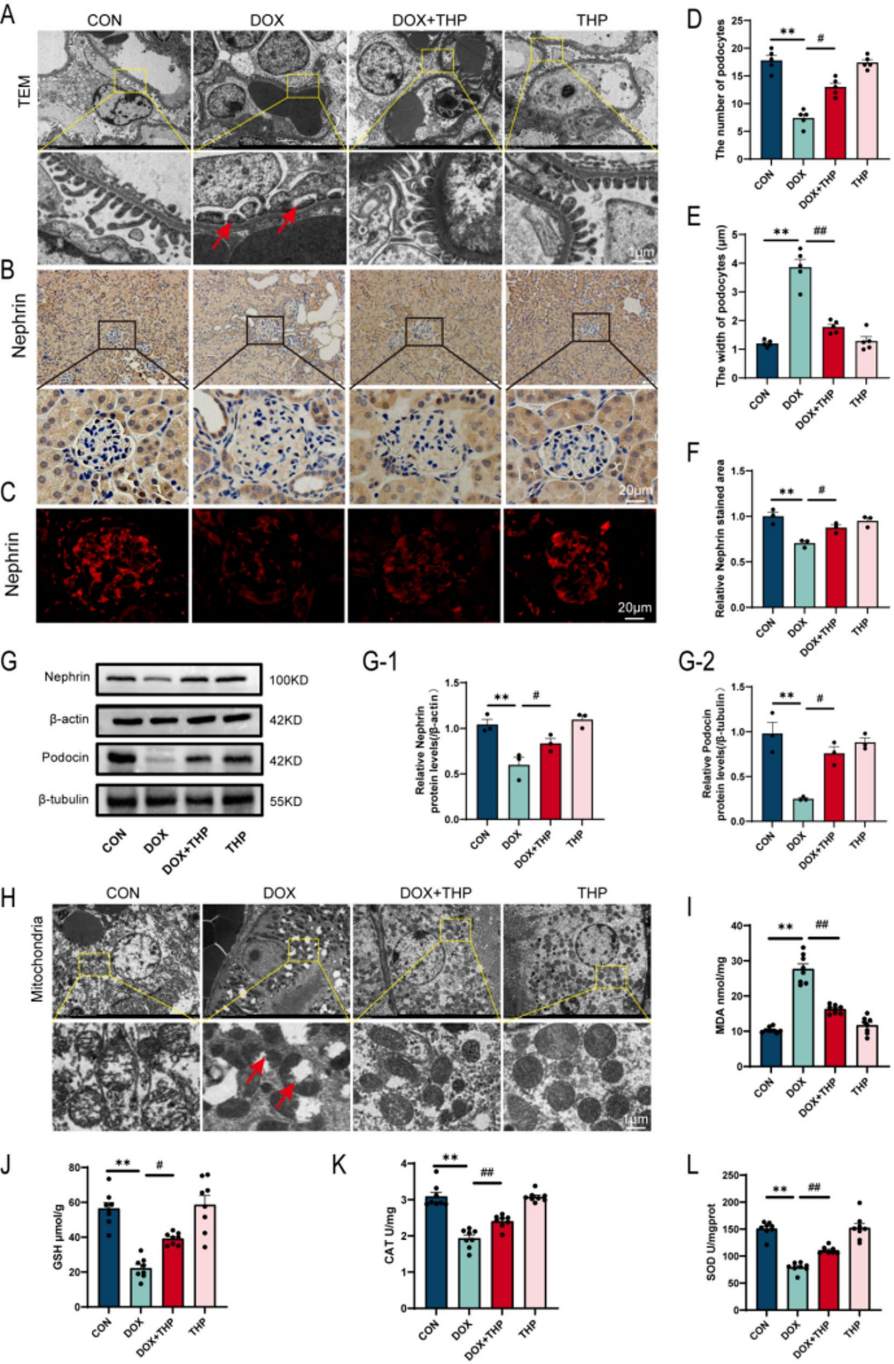
THP attenuates DOX-induced podocyte injury and mitochondrial function in mice. (**A**) Transmission electron microscopy (TEM) images showing representative foot process morphology in different groups. Scale bar = 1 μm. (**B**) Immunohistochemical detection of the podocyte marker protein Nephrin, Upper: 100×, scale bar = 50 μm; Lower insets: 400×, scale bar = 20 μm. (**C**) Immunofluorescence staining for Nephrin, Scale bar = 20 μm. (**D**-**E**) Quantitative analysis the number and width of podocyte in A image (*n* = 5). (**F**) Immunohistochemical staining quantification of Nephrin in each group (*n* = 3). (**G**) Western blot analysis of Nephrin and Podocin protein expression levels and semiquantitative analysis of them (G1-G2) (*n* = 3). (**H**) Transmission electron microscopy images reveal mitochondrial morphology in podocytes of renal tissue. Red arrows indicate mitochondrial damage, including swelling, rupture, and loss of cristae. Scale bar = 1 μm. (**I**-**L**) Levels of MDA, GSH, CAT, and SOD in the kidney of different groups (*n* = 8). Data are presented as mean ± SEM. **P < 0.01, vs. control group; ^#^*P* < 0.05, ^##^*P* < 0.01 vs. DOX group

Podocytes play a crucial role in maintaining the glomerular filtration barrier, regulating filtration function, and preserving glomerular structural stability [34]. Therefore, we investigated whether THP’s renoprotective effects were mediated by preserving podocyte integrity. Transmission electron microscopy (Fig. 2A) revealed that DOX treatment led to fusion and a reduction in podocyte foot processes (Fig. 2D-E), indicating impaired podocyte morphology and function (*P* < 0.01). In contrast, THP treatment significantly increased the number and length of podocyte foot processes and restored their orderly arrangement (*P* < 0.05). Moreover, immunohistochemical and immunofluorescence analyses (Fig. 2B-C) demonstrated that Nephrin expression, a podocyte-specific protein, was markedly reduced in the DOX group (*P* < 0.01), a decrease that was reversed by THP treatment (*P* < 0.01). Western blot (WB) analysis (Fig. 2G-G2) further confirmed that DOX notably decreased Nephrin and Podocin expression (*P* < 0.01), while THP treatment significantly upregulated their expression levels. The observations indicated that THP preserved the structural and functional integrity of the glomerular filtration barrier by enhancing podocyte-specific protein expression, ultimately reducing DOX-induced proteinuria and podocyte damage.

To evaluate the effects of DOX on oxidative stress, mitochondrial ultrastructure in the kidney tissues was examined using electron microscopy. In the DOX group, mitochondria exhibited swelling, rupture, and cristae loss, indicative of severe structural damage compared with controls (Fig. 2H). Mitochondrial dysfunction has been reported to impair ATP production and increase levels of oxidative stress in the body [35]. Notably, THP treatment partially restored mitochondrial morphology and function. We further quantified oxidative stress markers (MDA, GSH, CAT, and SOD) using commercial assay kits (Fig. 2I-L). Compared with controls, DOX-treated mice exhibited a significant increase in MDA levels (*P* < 0.01) and marked reductions in GSH, CAT, and SOD activities (*P* < 0.01). Following THP treatment, MDA levels significantly decreased (*P* < 0.01), while GSH, CAT, and SOD activities markedly increased (*P* < 0.05). The evidence demonstrated that THP alleviated DOX-induced oxidative stress and partially restores mitochondrial integrity and function.

### THP reduced DOX-induced renal inflammation and regulated apoptosis in mice

**Fig. 3:**
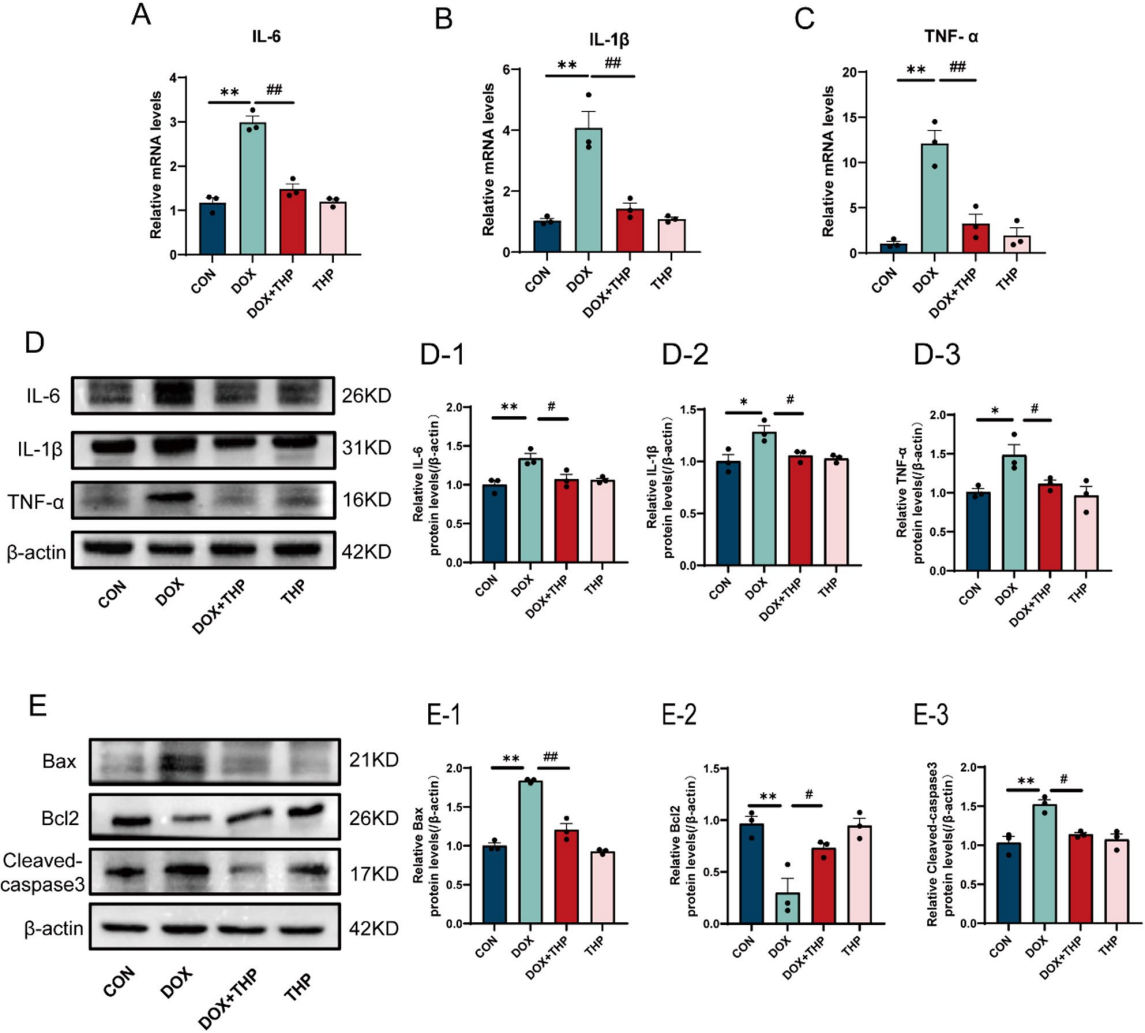
THP alleviates DOX-induced inflammation and apoptosis in the kidney of mice. (**A**-**C**) RT-qPCR analysis of IL-6, IL-1β, and TNF-α mRNA levels in different groups. (**D**) Western blot analysis of IL-6, IL-1β, and TNF-α protein expression levels and semiquantitative analysis of them (D1-D3), (*n* = 3). (**E**) Western blot analysis of Bax, Bcl2, and Cleaved-caspase3 protein expression levels and semiquantitative analysis of them (E1-E3), (*n* = 3). Data are presented as mean ± SEM. *P < 0.05, **P < 0.01, vs. control group; ^#^*P* < 0.05, ^##^*P* < 0.01 vs. DOX group

Next, we assessed the expression of inflammatory markers in the kidney tissues using RT-qPCR and Western blot analysis. RT-qPCR results (Fig. 3A-C) showed a marked increase in the mRNA levels of pro-inflammatory cytokines, such as IL-6, IL-1β, and TNF-α, in the DOX group compared to the control group (*P* < 0.001). THP treatment notably downregulated the mRNA levels of these cytokines (*P* < 0.01). In parallel, western blot analysis (Fig. 3D-D3) further confirmed a significant increase in the protein levels of IL-6, IL-1β, and TNF-α in the DOX group compared to the control group (*P* < 0.05). THP treatment markedly alleviated this inflammatory response, as evidenced by the reduced cytokine expression (*P* < 0.05). The evidence indicated that THP exerted its kidney-protecting role, in part by suppressing the inflammatory response.

Subsequently, we investigated the effect of DOX on apoptosis signaling in vivo by examining proteins involved in apoptosis regulation through Western blot analysis. Figure 3E-E3 demonstrate a significant increase in the protein levels of pro-apoptotic factors Bax and cleaved-Caspase3 in the kidney tissues of the DOX group compared with the normal group (*P* < 0.01), while the expression of the anti-apoptotic factor Bcl2 was significantly decreased (*P* < 0.01). However, following THP treatment, the expression levels of Bax and cleaved-Caspase3 were significantly 4downregulated (*P* < 0.01), whereas Bcl2 expression was markedly upregulated (*P* < 0.05). Based on these findings, we demonstrated that THP mitigated DOX-induced kidney damage by inhibiting apoptotic signaling pathways.

### THP attenuated DOX-induced renal injury in mice through Sirt3-mediated Nrf2/HO-1 signaling pathway

**Fig. 4:**
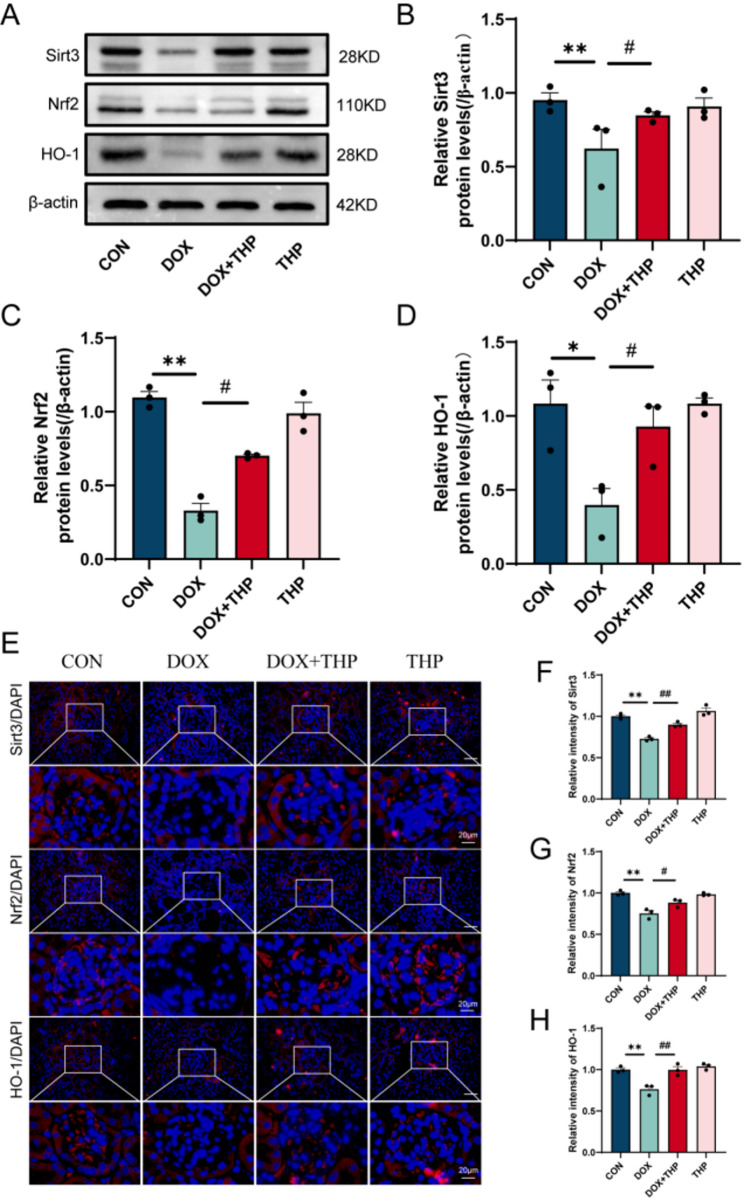
THP exerts renal protective effects by activating Sirt3-mediated Nrf2/HO-1 signaling pathway. (**A**) Western blot analysis of Sirt3, Nrf2, and HO-1 protein expression in the kidney of different groups. (**B**-**D**) Semiquantification of Sirt3, Nrf2, and HO-1 protein levels. (**E**) Immunofluorescence staining of Sirt3, Nrf2 and HO-1 in glomerulus of different groups. Upper: 100×, scale bar = 50 μm; Lower insets: 400×, scale bar = 20 μm. (**F**-**H**) Quantification of fluorescence intensity for Sirt3, Nrf2, and HO-1. Data are presented as mean ± SEM (*n* = 3). *P < 0.05, **P < 0.01, vs. control group; ^#^*P* < 0.05, ^##^*P* < 0.01vs. DOX group

To elucidate the mechanism underlying THP’s renoprotective effects, we hypothesized that THP activates Sirt3-mediated Nrf2/HO-1 signaling cascade. Western blot analysis (Fig. 4A-D) demonstrated that the protein expression levels of Sirt3, Nrf2, and HO-1 were markedly reduced in the DOX group relative to the controls (*P* < 0.05), indicating that DOX inhibited this pathway. In contrast, treatment with THP significantly upregulated the expression of these proteins (*P* < 0.05). Consistently, immunofluorescence analysis (Fig. 4E-H) confirmed these findings: fluorescence intensities for Nrf2, Sirt3, and HO‑1 were markedly lower in the DOX group (*P* < 0.01) but significantly increased following THP administration (*P* < 0.05). The research outcomes supported the hypothesis that THP exerted its nephroprotective effects by activating the Sirt3/Nrf2/HO-1 signaling cascade.

### THP attenuates DOX-induced podocyte injury through Sirt3-mediated Nrf2/HO-1 signaling pathway

**Fig. 5:**
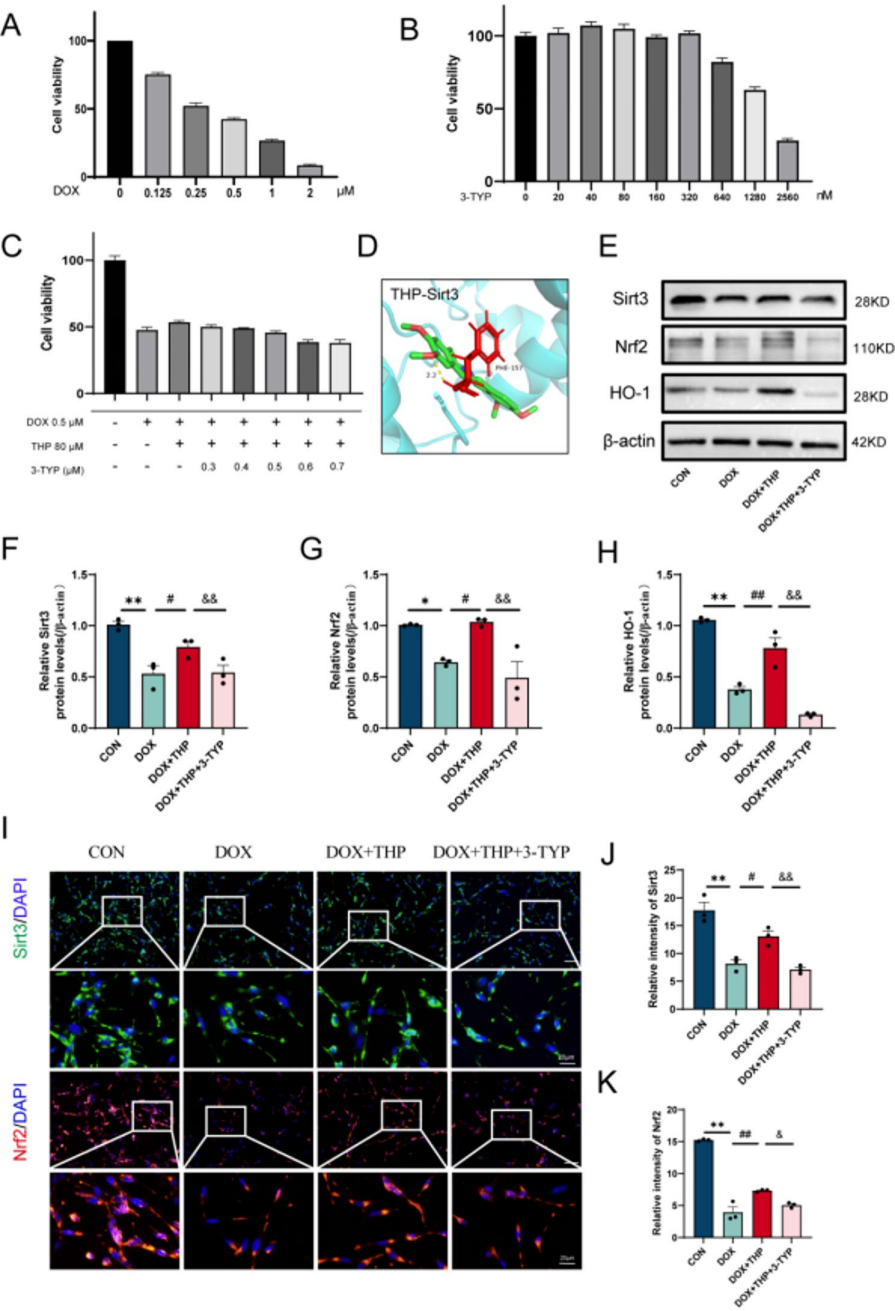
THP inhibits DOX-induced podocyte injury via Sirt3-mediated Nrf2/HO-1 signaling pathway. (**A**) Cell viability of MPC-5 cells treated with DOX. (**B**) Cell viability of MPC-5 cells treated with the Sirt3 inhibitor 3-TYP. (**C**) Cell viability of MPC-5 cells treated with DOX, THP, 3-TYP. (**D**) Molecular docking between THP and Sirt3. (**E**) Western blot analysis of Sirt3, Nrf2, and HO-1 protein expression in MPC-5 cells. (**F**-**H**) Semiquantification of Sirt3, Nrf2, and HO-1 protein levels. (**I**) Immunofluorescence staining for Sirt3, Nrf2, and HO-1. Upper: scale bar = 100 μm; Lower insets: scale bar = 20 μm. (**J**-**K**) Quantification of fluorescence intensity for Sirt3 and Nrf2. Data are presented as mean ± SEM (*n* = 3). *P < 0.05, **P < 0.01, vs. control group; ^#^*P* < 0.05, ^##^*P* < 0.01vs. DOX group; ^&^*P* < 0.05, ^&&^*P* < 0.01 vs. DOX + THP+3-TYP group

Finally, cellular experiments using MPC‑5 cells provided further evidence for the involvement of the Sirt3-mediated Nrf2/HO-1 signaling pathway in THP-mediated renoprotection. We consulted extensive literature and combined the CCK-8 assay to determine the optimal concentrations of DOX and the Sirt3 inhibitor 3-TYP (Fig. 5A-C). Molecular docking analysis (Fig. 5D) revealed that THP has a high binding affinity with the active pocket of Sirt3 (docking energy: -6.91 kcal/mol), and can form hydrogen bonds and hydrophobic interactions with the functionally crucial residue PHE-157, suggesting that Sirt3 may be a potential target for THP. To verify the specific regulatory relationship between THP and Sirt3 (rather than Sirt1/2), and to confirm the role of the Sirt3-specific inhibitor 3-TYP in this process, we examined the mRNA expression levels of Sirt1, Sirt2, and Sirt3 via PCR. The results showed (Supplementary Figure S1) that 3-TYP treatment significantly downregulated the mRNA expression of Sirt3, Sirt2, and Sirt1 *(P* < 0.01). Notably, THP potently reversed the 3-TYP-induced suppression of Sirt3 (*P* < 0.01), exerted no significant effect on Sirt2 expression (ns), and only weakly reversed the inhibition of Sirt1 (*P* < 0.05). These findings indicate that THP exhibits a specific regulatory association with Sirt3. Consistently, Western blot analysis (Fig. 5E–H) showed that DOX significantly reduced the protein expression levels of Sirt3, Nrf2, and HO‑1 in MPC‑5 cells (*P* < 0.05), whereas THP treatment reactivated the pathway and increased their expression (*P* < 0.05). Notably, the addition of 3‑TYP in the DOX + THP+3‑TYP group re-inhibited the Sirt3-mediated Nrf2/HO-1 signaling pathway compared with the DOX + THP group (*P* < 0.01). These trends were further corroborated by immunofluorescence analysis (Fig. 5I–K). Collectively, the research findings confirmed that THP mitigates DOX‑induced podocyte injury, at least in part, by activating the Sirt3-mediated Nrf2/HO-1 signaling pathway.

## Discussion

Doxorubicin, as a broad-spectrum anthracycline-based chemotherapeutic agent, has irreplaceable clinical value in the treatment of a variety of malignancies, including breast cancer and lymphoma [36]. However, its dose-dependent systemic toxicity severely limits its long-term application, with cardiotoxicity being of greatest concern and nephrotoxicity being easily overlooked [37]. Although animal models confirm that DOX induces podocyte apoptosis, oxidative damage to renal tubular epithelium and interstitial fibrosis, the molecular mechanisms remain controversial [38, 39]. Notably, existing nephroprotective agents (e.g., hydration therapy or antioxidants) are difficult to balance efficacy and safety due to the lack of targeting of DOX-specific toxicological mechanisms [40]. Therefore, by exploring the renoprotective mechanism of THP, this paper aims to identify a potential drug that can block nephrotoxicity while preserving its anticancer activity. In this study, we selected male BALB/c mice and successfully established a doxorubicin-induced nephropathy model by administering a 10 mg/kg dose of doxorubicin via tail vein injection. We employed BALB/c inbred mice to construct the DOX-induced nephropathy model, given their uniform genetic background, high susceptibility to DOX-induced renal injury with typical pathological phenotypes, high homology of renal tissue structure to humans, and wide use in renal injury toxicological research with solid literature support. Subsequently, we evaluated renal function-related indices through serum and urine analysis, revealing that doxorubicin not only induced significant proteinuria but also triggered inflammatory responses, oxidative stress, and apoptosis signaling in the kidney. Based on these findings, we hypothesize that doxorubicin-induced renal injury in mice is mediated through inflammation, oxidative stress, and apoptosis pathways.

**Fig. 6:**
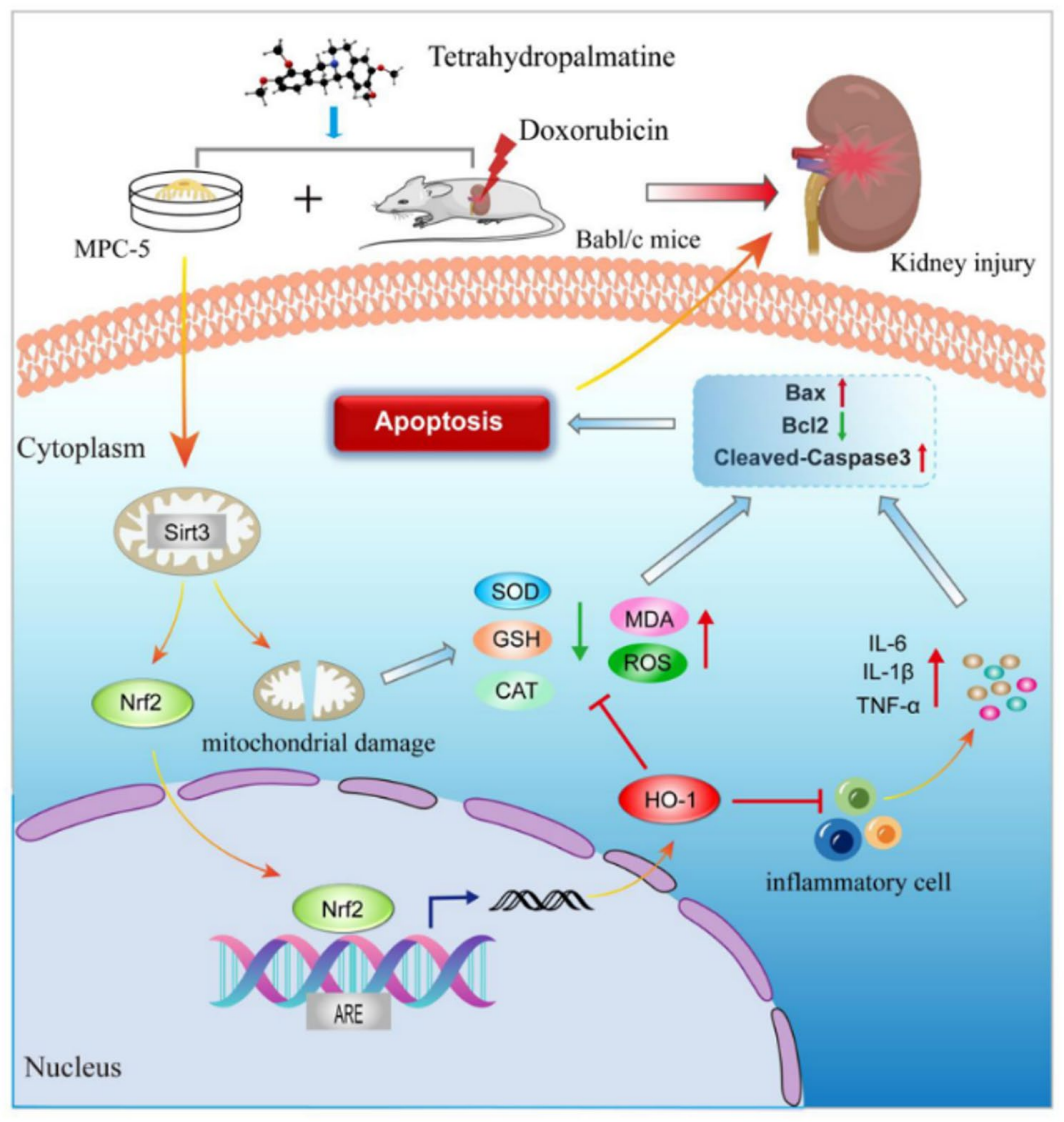
Mechanism diagram

Sirt3 is a mitochondrial NAD-dependent deacetylase that predominantly regulates the acetylation of proteins within the mitochondria [41]. Moreover, Sirt3 is crucial for modulating key biological processes such as inflammation, oxidative stress, apoptosis, and DNA damage, all of which are closely linked to the development of renal disease [42]. It has been suggested that Sirt3 maintains mitochondrial homeostasis by facilitating peroxisome-mitochondrial interactions through peroxisome biogenesis factor 5 (PEX5) [43], which is consistent with our findings. In our study, THP enhanced mitochondrial morphology and function via activation of the Sirt3 signaling pathway, thereby inhibiting oxidative stress in vivo and reducing MDA levels (Fig. 6).

Additionally, previous studies have demonstrated that Sirt3 is crucial for maintaining mitochondrial respiratory chain function [44] and mitochondrial autophagy [45], which helps mitigate disease progression by activating downstream pathways and preserving intracellular homeostasis. The Nrf2/HO-1 signaling pathway is a key endogenous antioxidant mechanism and an important target for inflammation-related diseases [46]. External factors can enhance Nrf2 activity, thereby regulating the transcription, modification, or expression of downstream proteins such as HO-1, GSH, and SOD [47]. Studies have shown that ferroptosis can be inhibited by activating the Nrf2/HO-1 signaling cascade, thereby alleviating diabetic kidney injury [48]. Moreover, activation of the Nrf2/HO-1 signaling cascade inhibits endoplasmic reticulum stress, further attenuating cellular pyroptosis and apoptosis, thus protecting the kidney from ischemia-reperfusion injury [49].

Literature review indicates a close association between Sirt3 and Nrf2 targets. Existing studies confirm that regulating the NRF2/SIRT3 axis can improve chondrocyte activity by inhibiting mitochondrial dysfunction [50]. Simultaneously, as an upstream regulatory molecule, Sirt3 enhances Nrf2 activity, thereby modulating fatty acid metabolism and cellular redox homeostasis [51]. To validate these mechanisms, this study employed the Sirt3-specific inhibitor 3-TYP. Results revealed that when Sirt3 expression was suppressed in cells, Nrf2’s antioxidant capacity significantly diminished. This mechanism may be related to reduced Nrf2 release following dissociation of the Nrf2/Keap1 complex. Molecular docking analysis revealed that THP has a high binding affinity with the active center of Sirt3, and can form hydrogen bonds and hydrophobic interactions with the functional key residue PHE-157, providing a structural basis for the interaction between the two. This suggests that Sirt3 may be a potential target for THP. Additionally, the in vitro and in vivo experiments in this study have confirmed that after inhibiting Sirt3, the activation of the Nrf2/HO-1 pathway and the renal protective effect of THP both significantly disappear, indicating that the biological function of THP is highly dependent on the activity of Sirt3. Our research demonstrates that THP activates Sirt3-mediated Nrf2/HO-1 signaling cascade to exert anti-inflammatory, antioxidant, and apoptosis-inhibiting effects in a doxorubicin-induced podocyte injury model.

Tetrahydropalmatine, a common active ingredient in traditional Chinese medicine derived from *Stephania cepharantha Hayata* and *Corydalis yanhusuo*, has primarily been studied for its analgesic, sedative, anti-addictive, and neuroprotective properties [22, 52]. However, its roles in anti-inflammatory, antioxidant, and anti-apoptotic processes have received less attention. As a result, there has been growing interest among researchers in investigating the impact of THP on additional target organs. For example, Prof. Chen’s team demonstrated that THP enhances the survival of skin flaps by regulating autophagy, reducing tissue edema, promoting angiogenesis, and mitigating apoptosis and oxidative stress [53]. Similarly, Prof. Dan Wu discovered that THP suppresses neuroinflammation through the NF-κB-NLRP3 inflammasome signaling cascade [54], while Prof. Wang’s group reported that THP inhibits ROS-induced activation of the NLRP3 inflammasome in MSU-triggered inflammatory responses [21]. Additionally, Prof. Han’s team observed that THP protects cardiomyocytes from acute myocardial ischemia due to its potent antioxidant and anti-apoptotic properties [23]. In our study, our results suggest that THP protects against DOX-induced nephropathy by alleviating oxidative stress, inflammation, and apoptosis. Specifically, we first examined mitochondrial morphology and function, followed by assessing changes in oxidative stress-related markers (MDA, GSH, CAT, and SOD) using commercially available kits. Our findings indicated that DOX notably decreased the levels of antioxidants (GSH, CAT, and SOD) and increased oxidized products (MDA). In the DOX nephropathy model group, the levels of inflammatory cytokines (IL-6, IL-1β, and TNF-α) in the kidney were found to be significantly higher compared to the control group. Furthermore, a decrease in the anti-apoptotic protein Bcl-2 was observed, while the levels of pro-apoptotic proteins Bax and Cleaved-Caspase-3 were found to be elevated. Interestingly, after administering THP for two weeks, we noted significant suppression of oxidative stress, inflammation, and apoptosis in the kidney, with some restoration of renal function.

Recent studies indicate that beyond its traditional pharmacological effects, THP’s anticancer activity is increasingly gaining attention among researchers. Studies have shown that in the field of hepatocellular carcinoma treatment, THP effectively reduces tumor growth in mouse HepG2 models through AMPK-mediated metabolic reprogramming [25] and can also kill HepG2 cells via autophagy-mediated metabolic switching without exerting adverse effects on other organs [55]. Additionally, THP has been found to enhance the cytotoxicity of CD8⁺ T cells against gastric cancer cells and potentiate the anti-tumor immune response [56]. In breast cancer BCa cells, THP blocks the transition from the G0/G1 phase to the S phase by promoting the degradation of estrogen receptor α (ERα) and inhibiting its transcriptional activity, leading to cell cycle arrest [57]. It has also been reported that THP can enhance the sensitivity of leukemia cells to DOX [58]. DOX, as a broad-spectrum anthracycline antitumor drug, has long seen its clinical application limited by side effects such as cumulative dose-dependent cardiotoxicity, nephrotoxicity, and myelosuppression, and our study confirms that THP can alleviate DOX-induced renal injury via the Sirt3-Nrf2/HO-1 signaling pathway. Based on the complementary nature of its toxicity mechanisms and THP’s pharmacological properties, we speculate that the combined application of THP and DOX might reduce the adverse effects of DOX monotherapy and enhance antitumor efficacy through synergistic effects. However, there is currently insufficient clinical data to support this hypothesis, necessitating further research for validation.

The glomerulus is the primary pathological site vulnerable to renal injury, and podocytes—key cells maintaining the structural integrity and functional stability of the glomerular filtration barrier—exhibit biological behaviors tightly linked to the progression of renal injury. Thus, the selection of podocytes for our in vitro experiments ensures strong representativeness and specificity. However, this study has several limitations: First, due to constraints in funding and technical feasibility, we did not employ gene knockout mouse models or siRNA interference strategies; instead, we validated our core conclusions via in vitro intervention with the Sirt3 inhibitor 3-TYP, complemented by molecular docking simulations and protein-level detection assays—findings that still hold considerable referential value. Second, 3-TYP is not a fully specific inhibitor and may cross-react with other Sirtuin family members (e.g., Sirt1, Sirt2). Additionally, inherent technical barriers in podocyte research, including their intrinsically low transfection efficiency (which hinders the performance of robust loss- or gain-of-function experiments such as Sirt3 siRNA knockdown or overexpression) and the challenges associated with constructing and delivering Sirt3 overexpression vectors in vivo (which requires targeted cellular uptake, sustained transgene expression, and minimal off-target effects in renal tissue), further limit the mechanistic depth of our current work. To uphold the rigor of our conclusions and address these research gaps, we propose a clear future research agenda: to generate stable Sirt3-overexpressing podocyte cell lines and Sirt3 transgenic animal models, and to combine these models with treatment using ML385—a selective Nrf2 inhibitor—to systematically dissect the hierarchical regulatory relationship within the Sirt3-Nrf2/HO-1 pathway, thereby providing definitive experimental evidence for the causal link between Sirt3 activation, Nrf2 nuclear translocation, and the attenuation of doxorubicin-induced renal injury.

## Conclusion

In summary, based on the in vivo and in vitro experiments and molecular docking analysis in this study, THP exerts a renoprotective effect, which may be attributed to its modulation of the Sirt3-mediated Nrf2/HO-1 signaling pathway. THP exhibits considerable potential as a therapeutic candidate for drug-induced renal injury; however, current research on this agent remains relatively preliminary and fragmented. Therefore, the precise molecular regulatory mechanisms of THP, as well as its in vivo efficacy and safety profiles, warrant further in-depth investigation.

## Supplementary Information

Below is the link to the electronic supplementary material.

Supplementary Material 1

Supplementary Material 2

## Data Availability

Data will be made available on request.

## Abbreviations

THP: Tetrahydropalmatine
DOX: Doxorubicin
UACR: Urine Albumin-to-Creatinine Ratio
mALB: Microalbumin
BUN: Blood Urea Nitrogen
GSH: Glutathione
SOD: Superoxide Dismutase
CAT: Catalase
MDA: Malondialdehyde
IL-6: Interleukin-6
IL-1β: Interleukin-1 beta
TNF-α: Tumor Necrosis Factor alpha
Bax: BCL2-Associated X Protein
Bcl-2: B-cell lymphoma 2
Sirt3: Sirtuin 3
Nrf2: Nuclear Factor Erythroid 2-Related Factor 2
HO-1: Heme Oxygenase-1
ROS: Reactive Oxygen Species
