# Supplementary material for: Tetrahydropalmatine may alleviate doxorubicin-induced renal injury by activating the Sirt3-mediated Nrf2/HO-1 pathway
Source: Biol Direct. 2026 Apr 30;21:113. doi: 10.1186/s13062-026-00773-9 (PMC13312763; doi:10.1186/s13062-026-00773-9)
Supplement: Supplementary file 1 — Supplementary Material 1 [file 13062_2026_773_MOESM1_ESM.pdf]

Supplementary Figure S1.

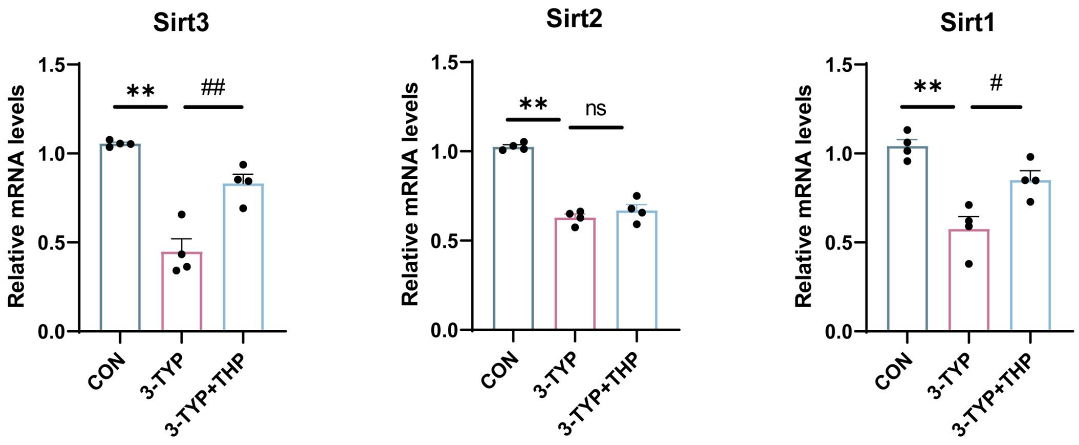

Sirt3, Sirt2, and Sirt1 mRNA relative expression levels in different groups in MPC-5 cells. ***P* < 0.01 vs. CON group; #*P* < 0.05, ##*P* < 0.01 vs. 3-TYP group.
