# Supplementary material for: Tetrahydropalmatine may alleviate doxorubicin-induced renal injury by activating the Sirt3-mediated Nrf2/HO-1 pathway
Source: Biol Direct. 2026 Apr 30;21:113. doi: 10.1186/s13062-026-00773-9 (PMC13312763; doi:10.1186/s13062-026-00773-9)
Supplement: Supplementary file 2 — Supplementary Material 2 [file 13062_2026_773_MOESM2_ESM.pdf]

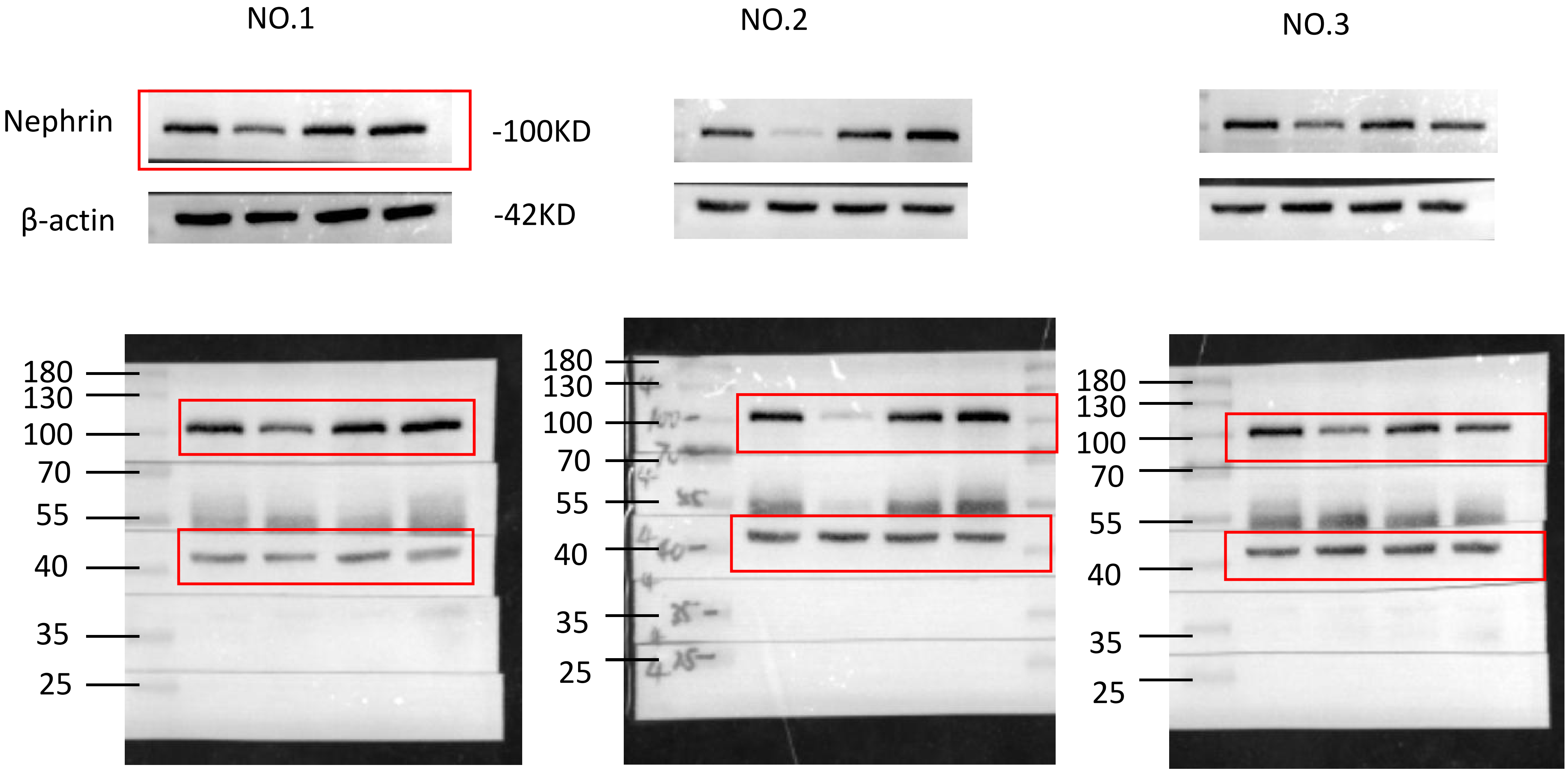
NO.1
NO.2
NO.3
Nephrin
β-actin
-100KD
-42KD
180
130
100
70
55
40
35
25
180
130
100
70
55
40
35
25
180
130
100
70
55
40
35
25

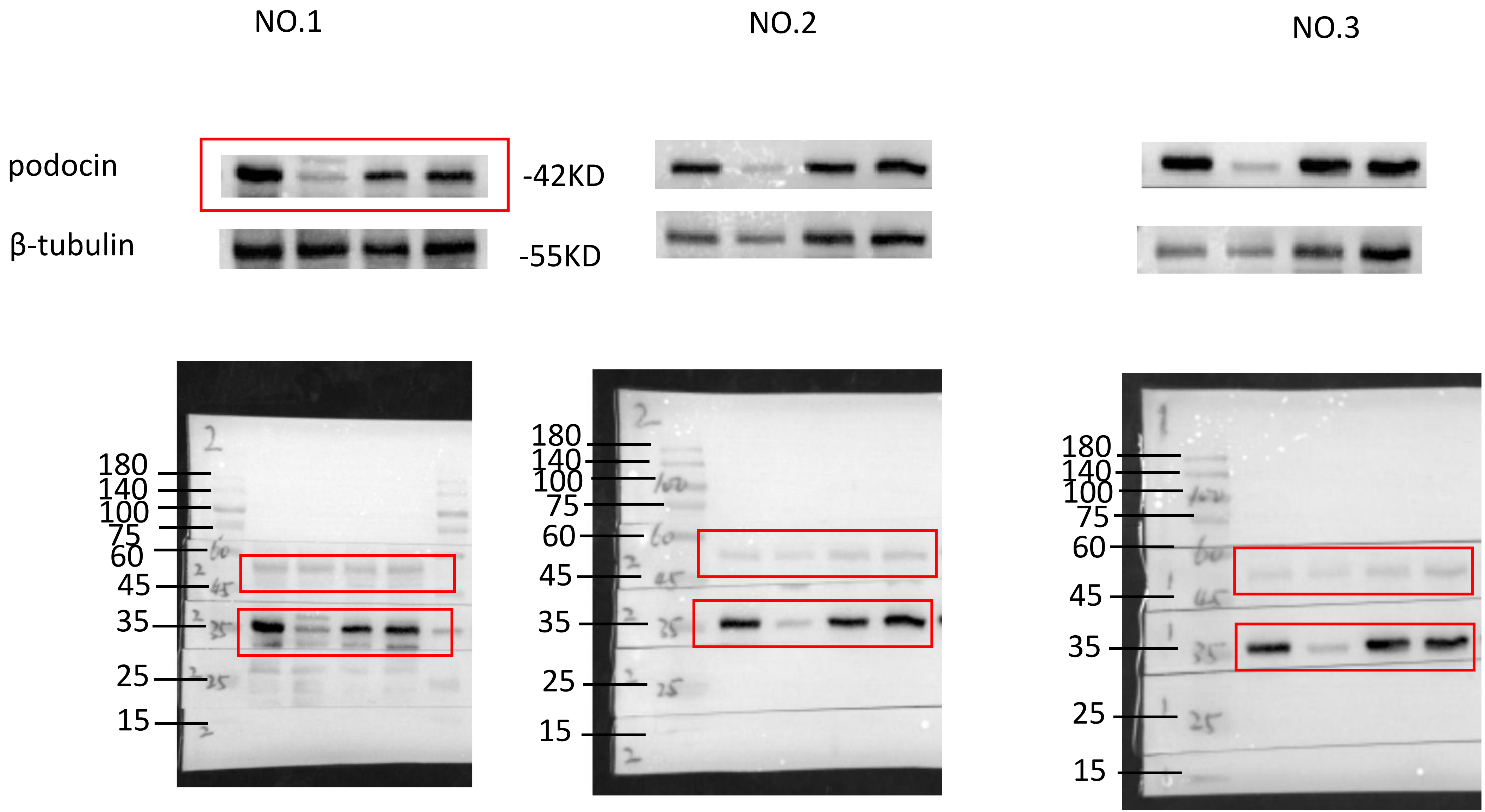
NO.1
NO.2
NO.3
podocin
-42KD
β-tubulin
-55KD
180
140
100
75
60
45
35
25
15

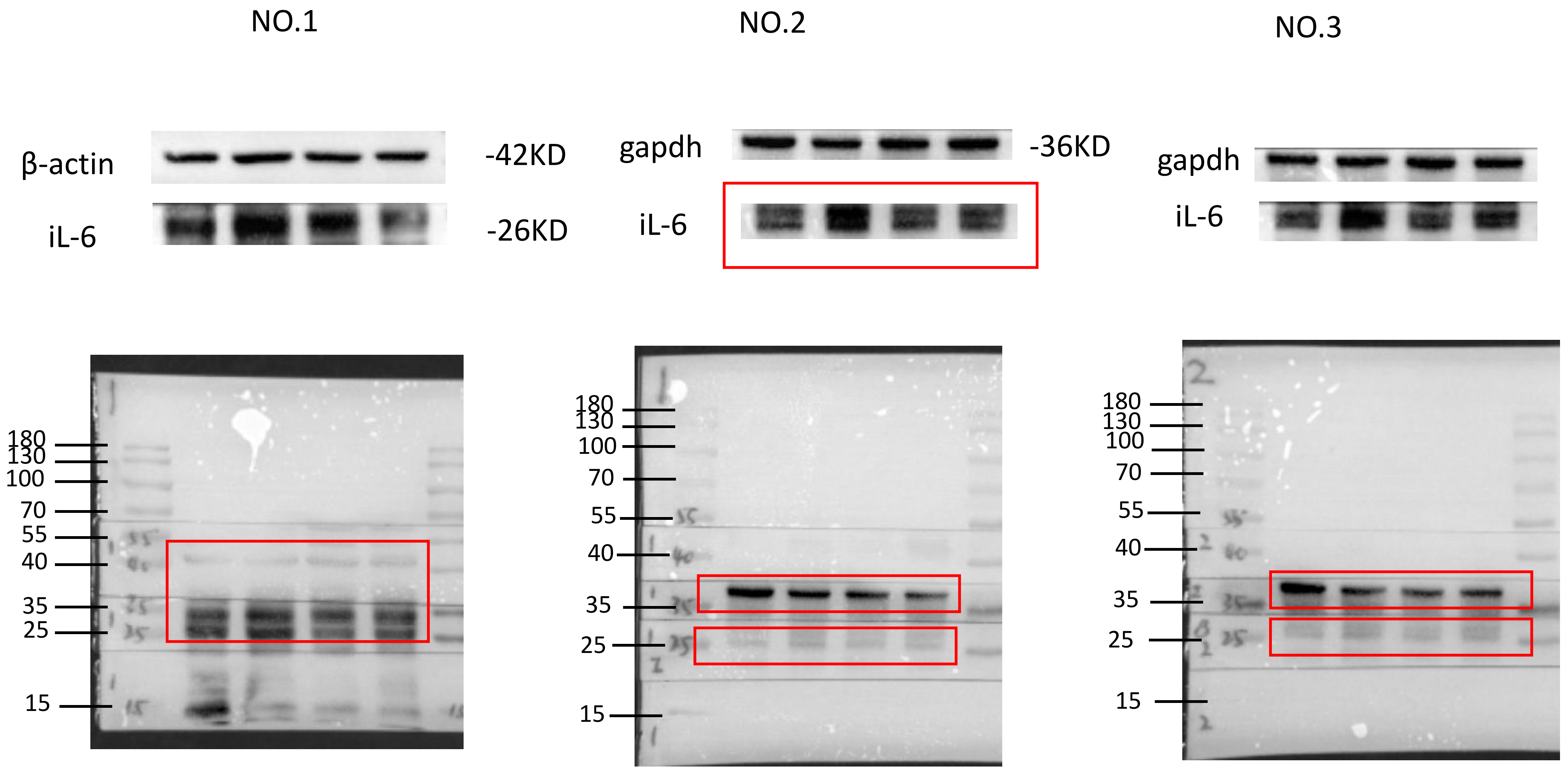
NO.1
NO.2
NO.3
β-actin
-42KD
iL-6
-26KD
gapdh
-36KD
iL-6
gapdh
iL-6
180
130
100
70
55
40
35
25
15
180
130
100
70
55
40
35
25
15
180
130
100
70
55
40
35
25
15

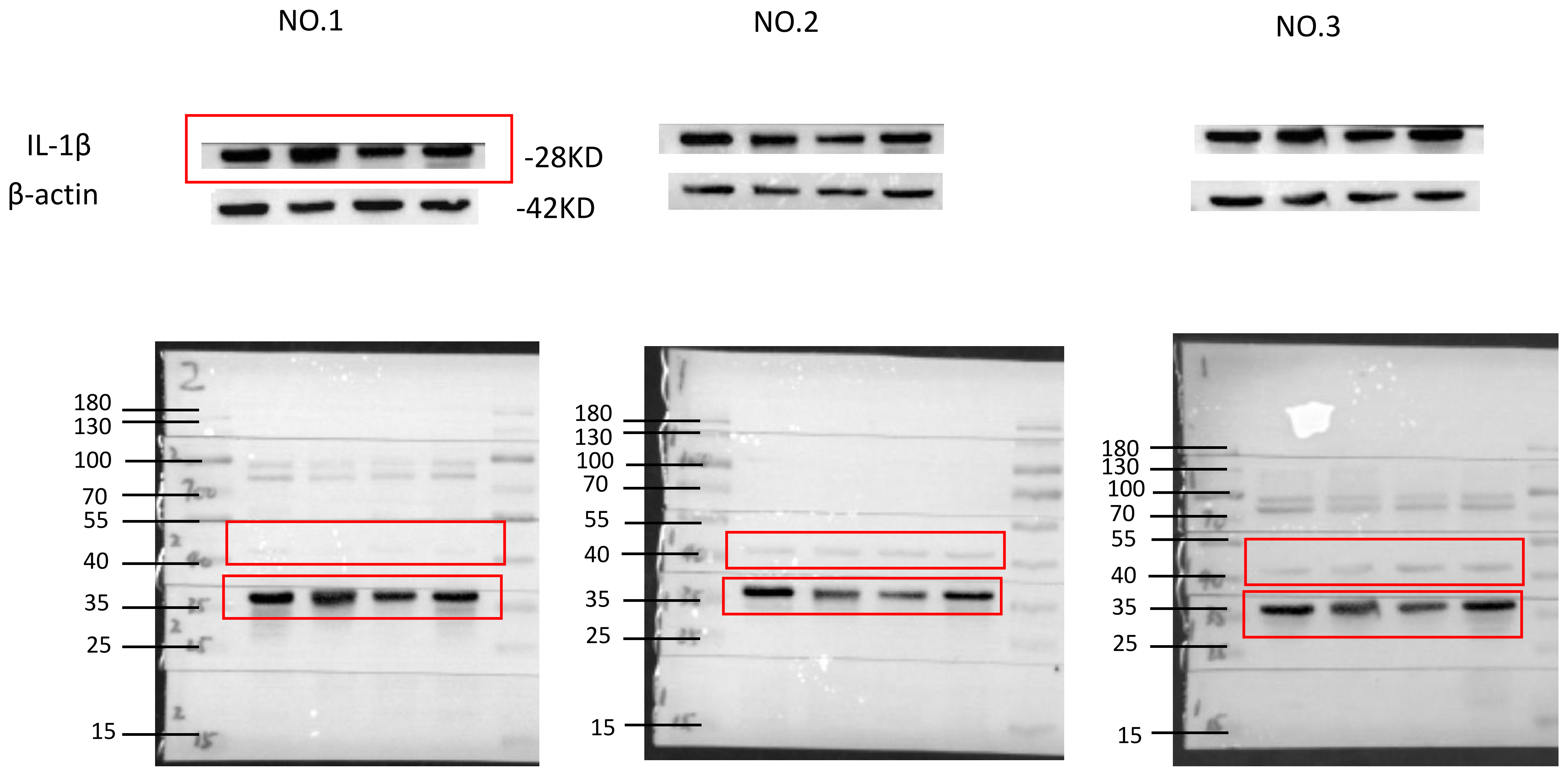
NO.1
NO.2
NO.3
IL-1β
β-actin
-28KD
-42KD
180
130
100
70
55
40
35
25
15

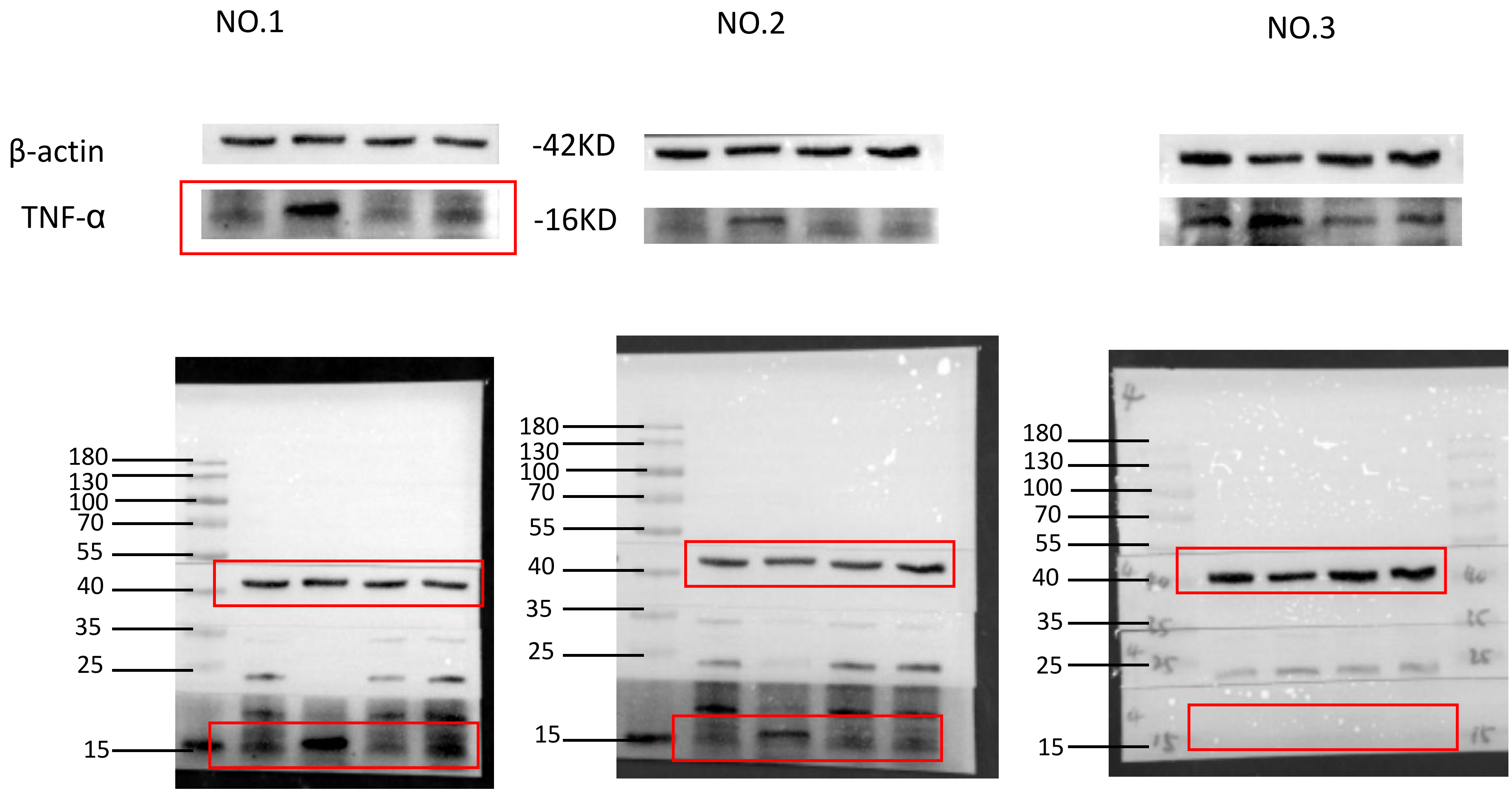

NO.1
NO.2
NO.3
β-actin
TNF-α
-42KD
-16KD
180
130
100
70
55
40
35
25
15

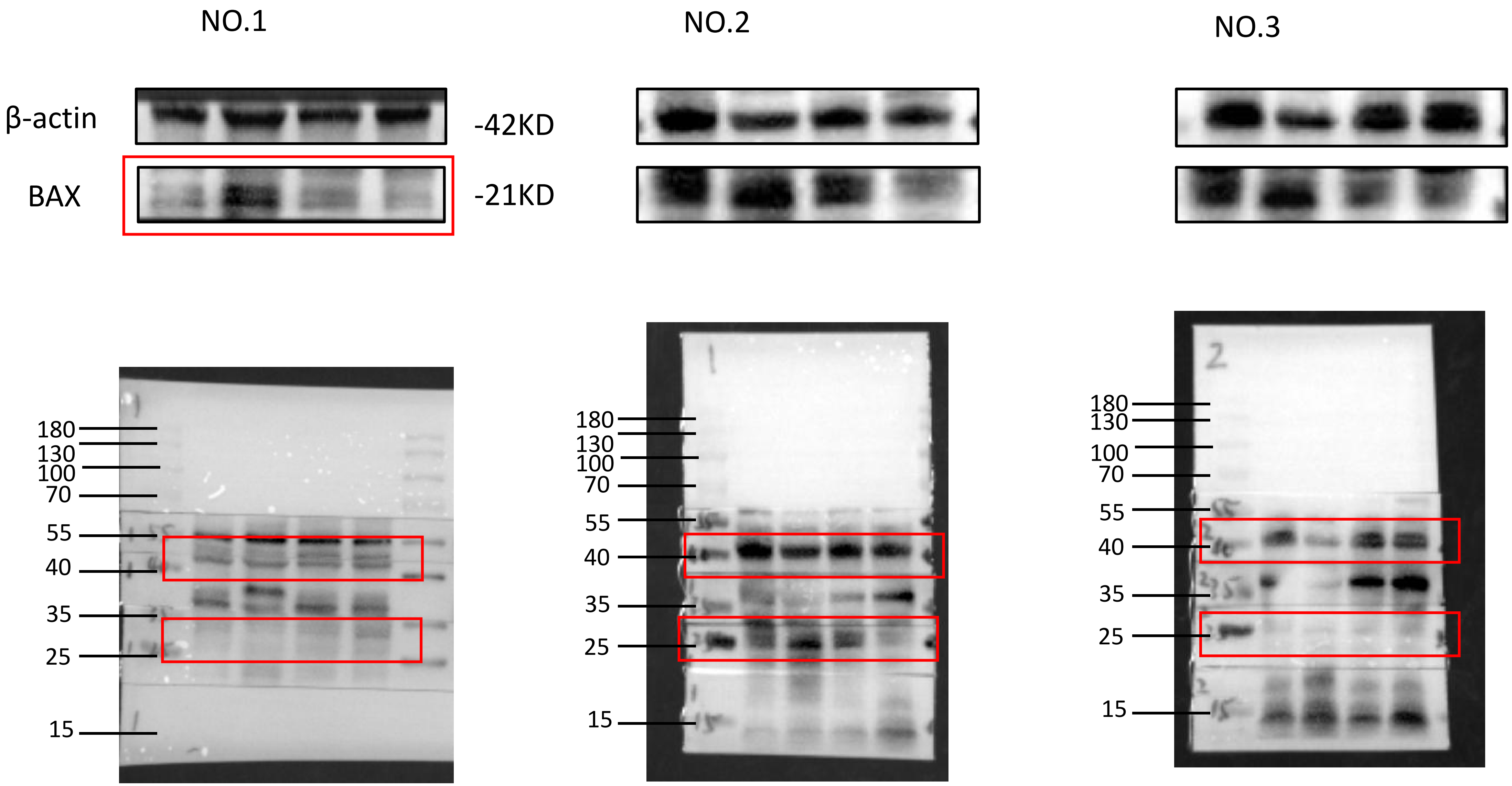
NO.1
NO.2
NO.3
β-actin
BAX
-42KD
-21KD
180
130
100
70
55
40
35
25
15

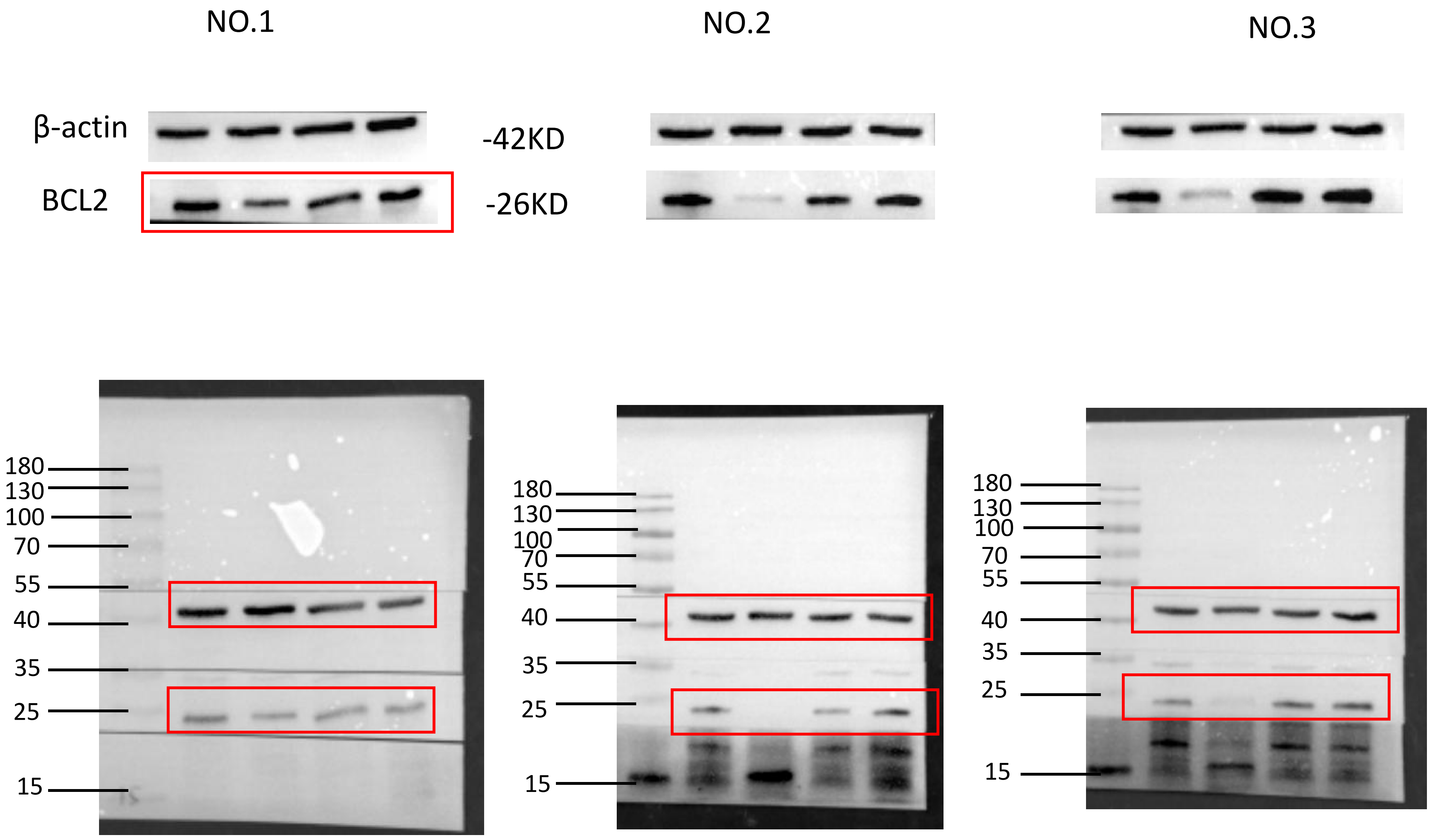
NO.1
NO.2
NO.3
β-actin
BCL2
-42KD
-26KD
180
130
100
70
55
40
35
25
15

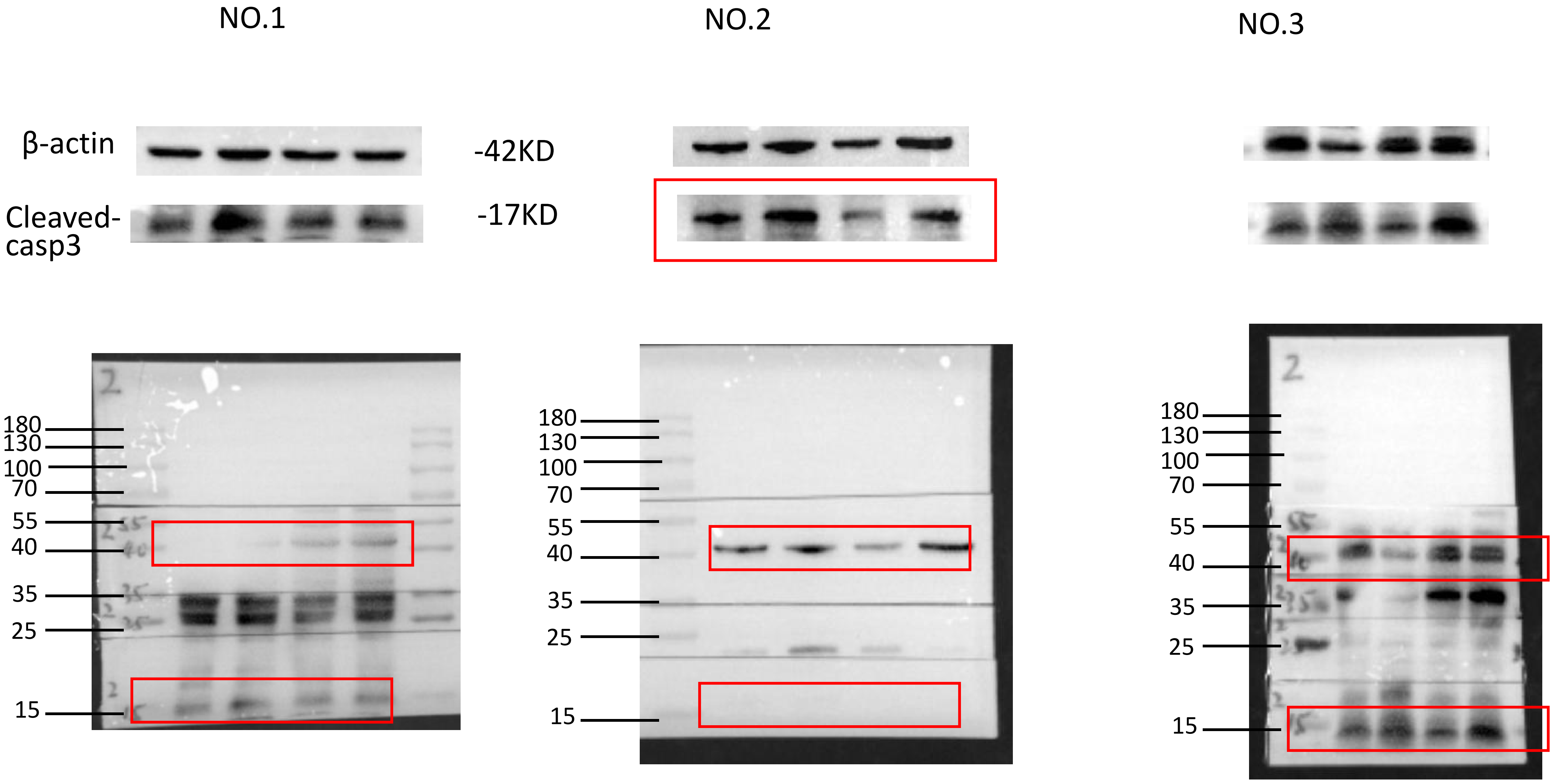
NO.1
NO.2
NO.3
β-actin
-42KD
Cleaved-casp3
-17KD
180
130
100
70
55
40
35
25
15

20240812

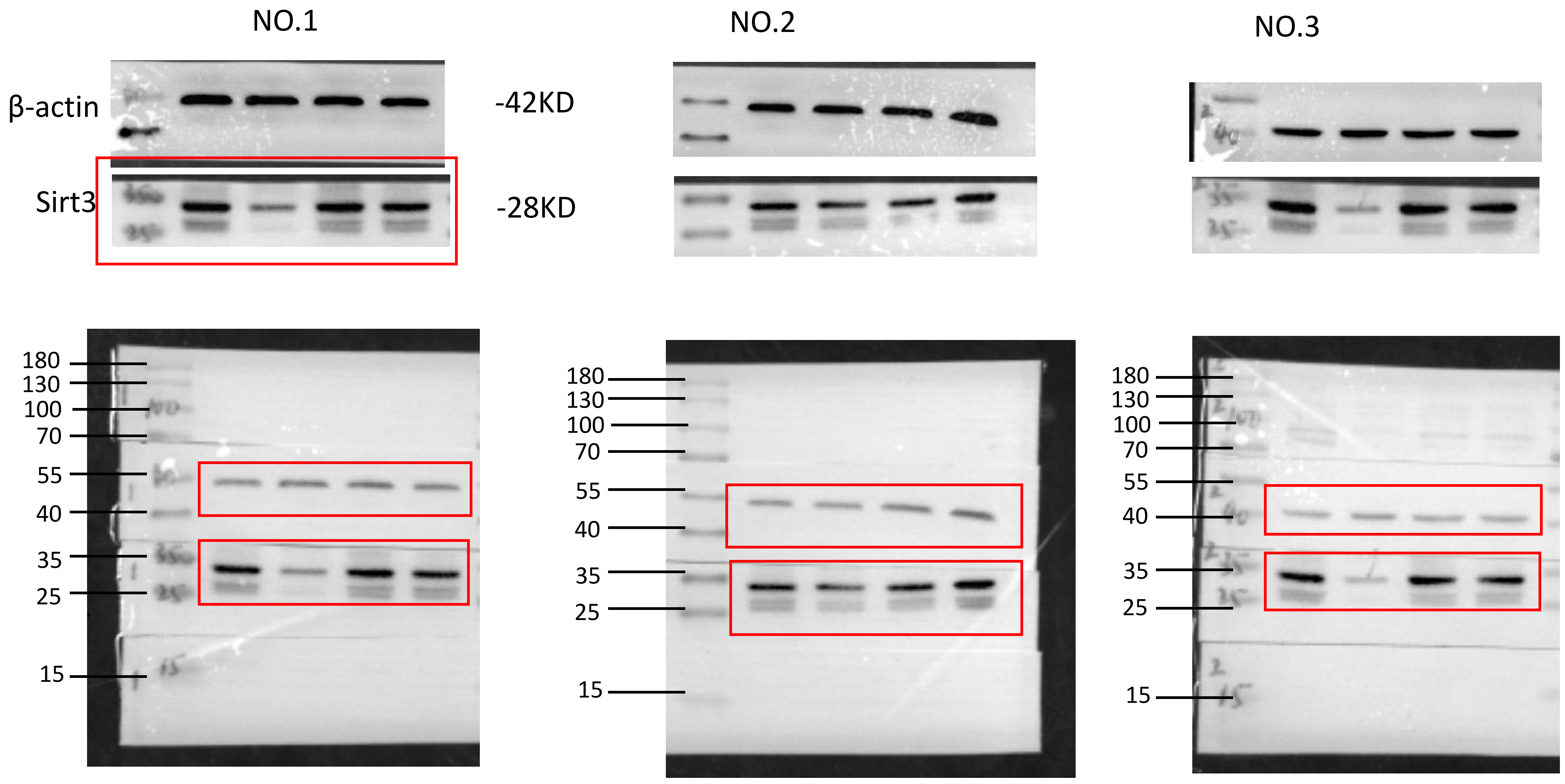
NO.1
NO.2
NO.3
β-actin
Sirt3
-42KD
-28KD
180
130
100
70
55
40
35
25
15

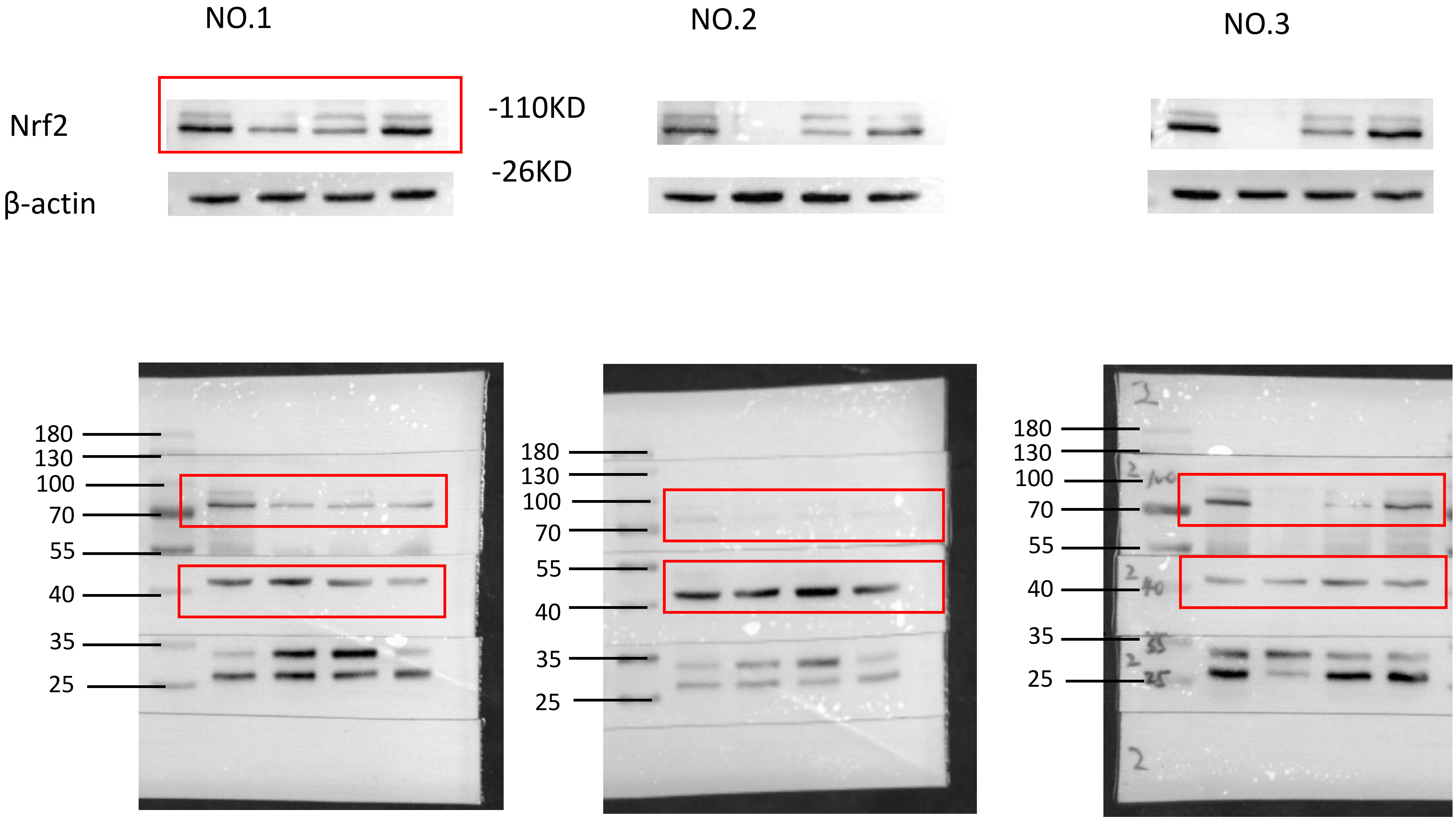

NO.1
NO.2
NO.3
Nrf2
β-actin
-110KD
-26KD
180
130
100
70
55
40
35
25

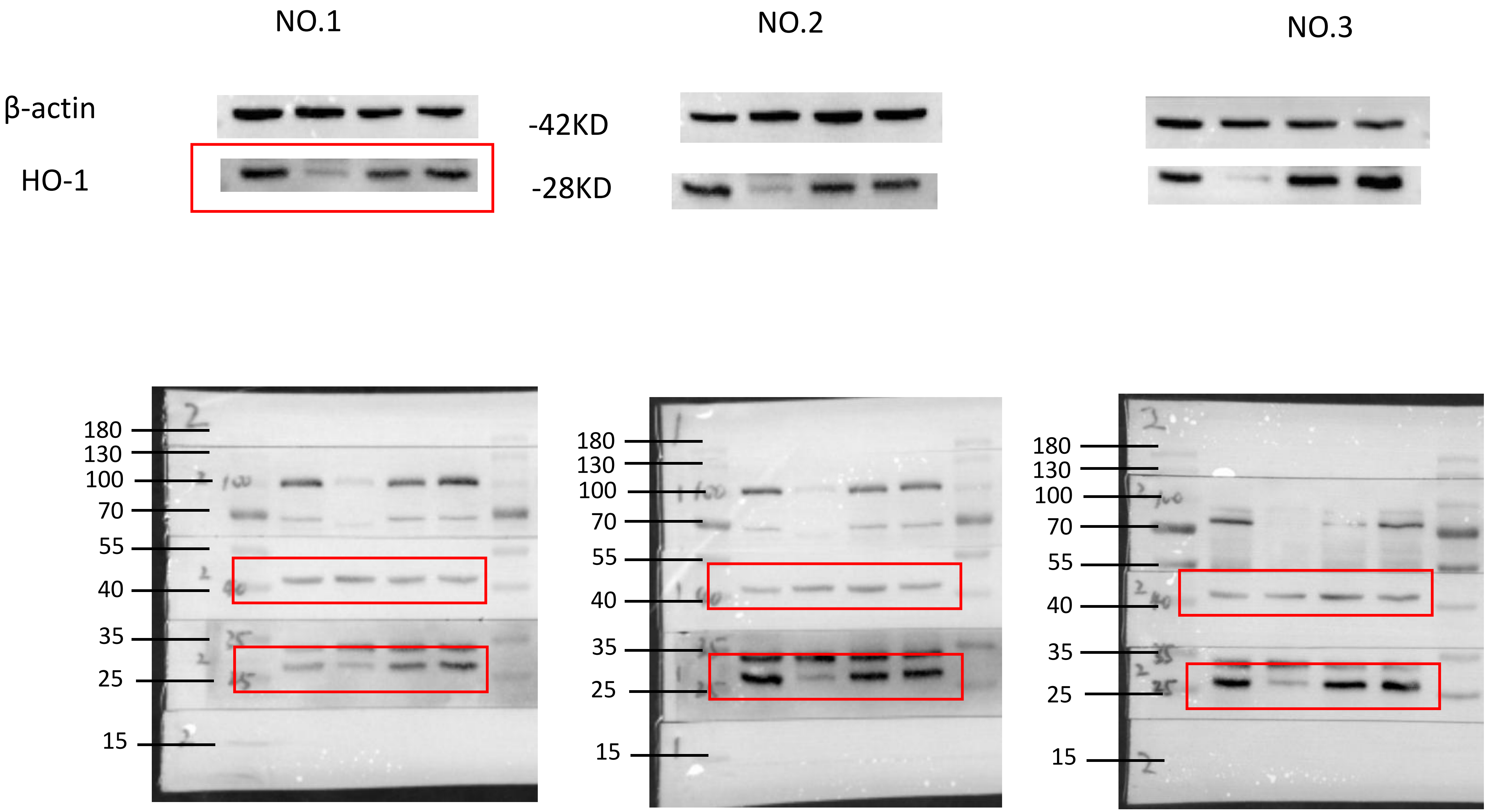
NO.1
NO.2
NO.3
β-actin
HO-1
-42KD
-28KD
180
130
100
70
55
40
35
25
15

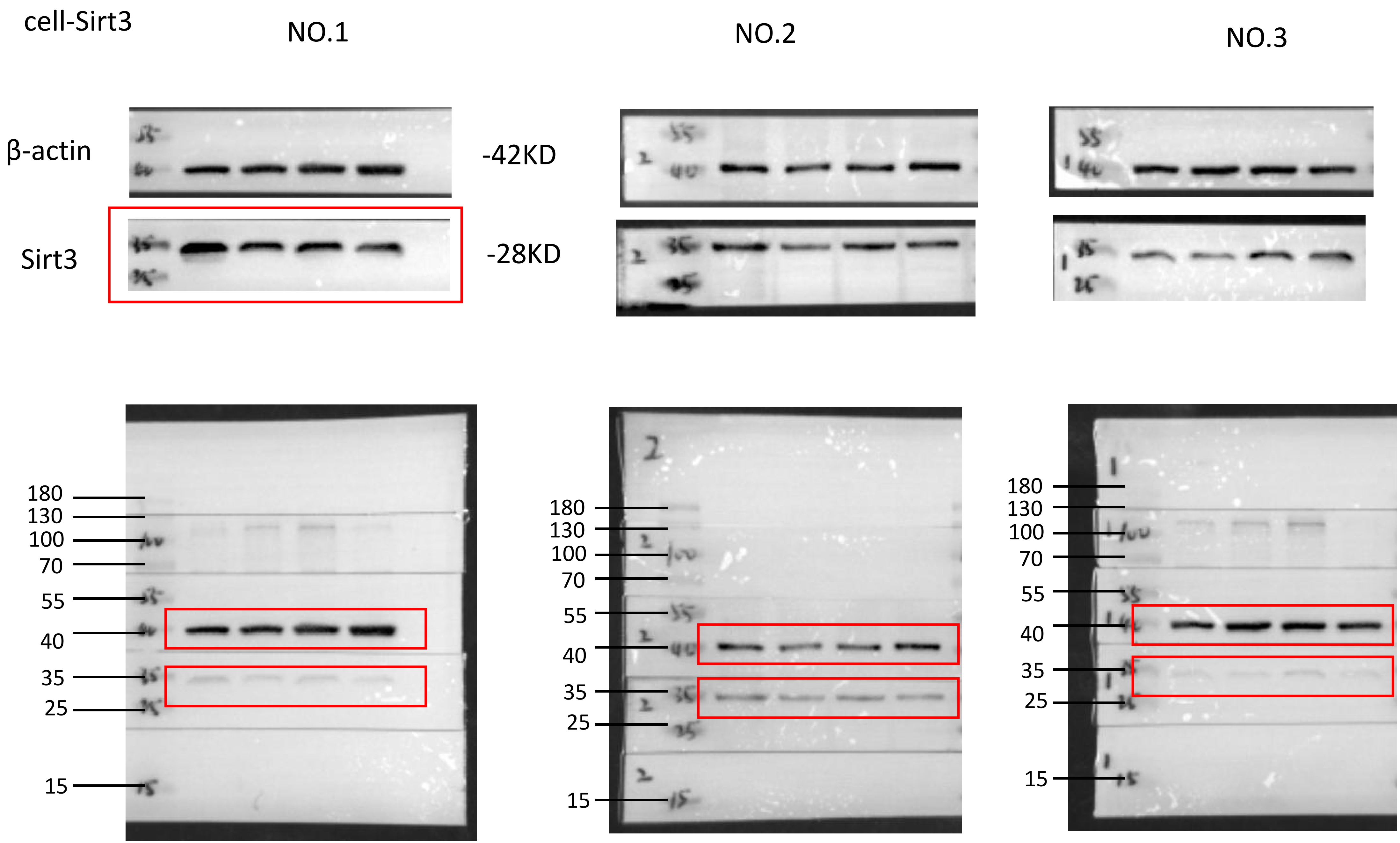
cell-Sirt3
NO.1
NO.2
NO.3
β-actin
-42KD
Sirt3
-28KD
180
130
100
70
55
40
35
25
15

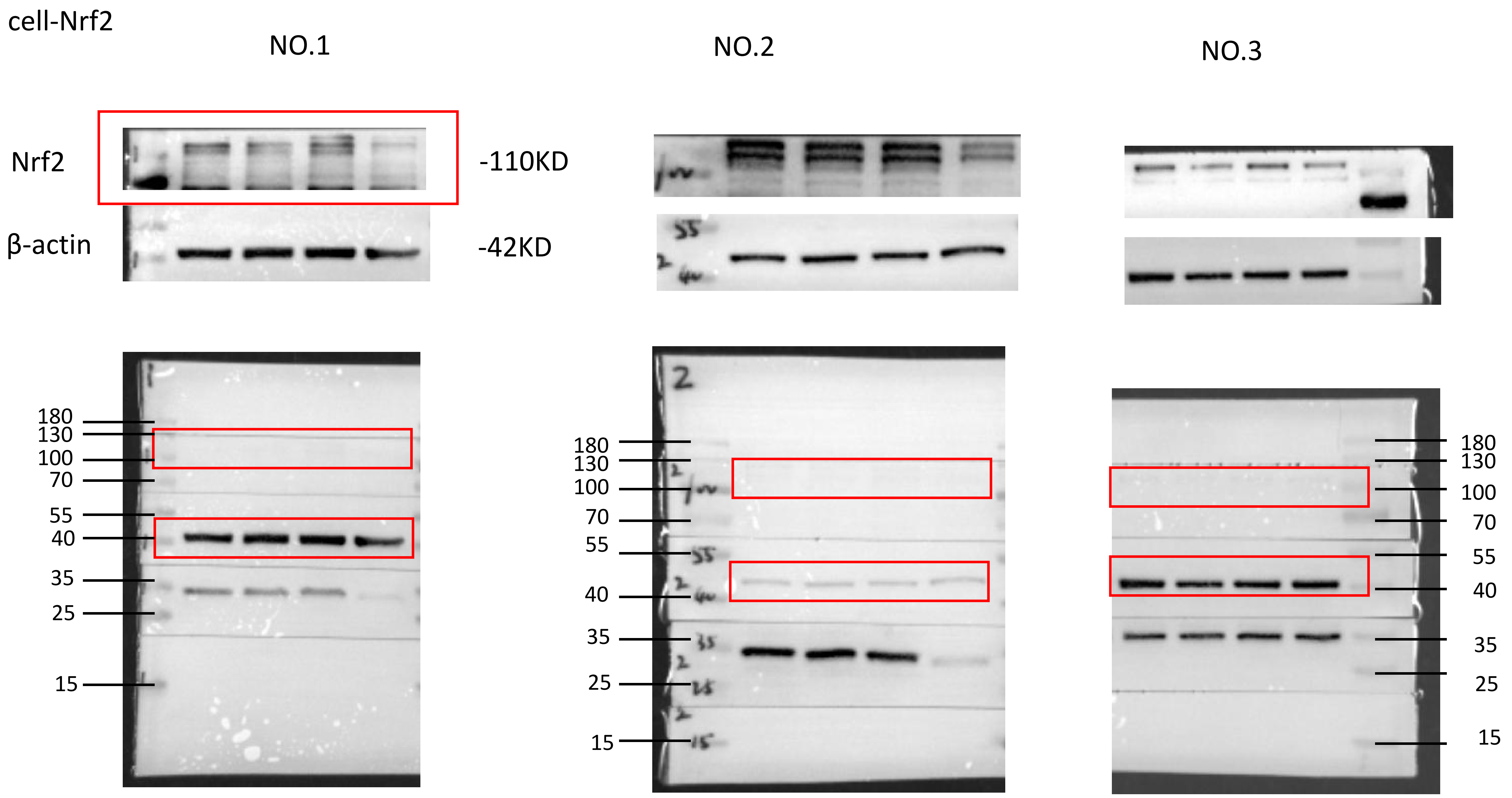
cell-Nrf2
NO.1
NO.2
NO.3
Nrf2
β-actin
-110KD
-42KD
180
130
100
70
55
40
35
25
15

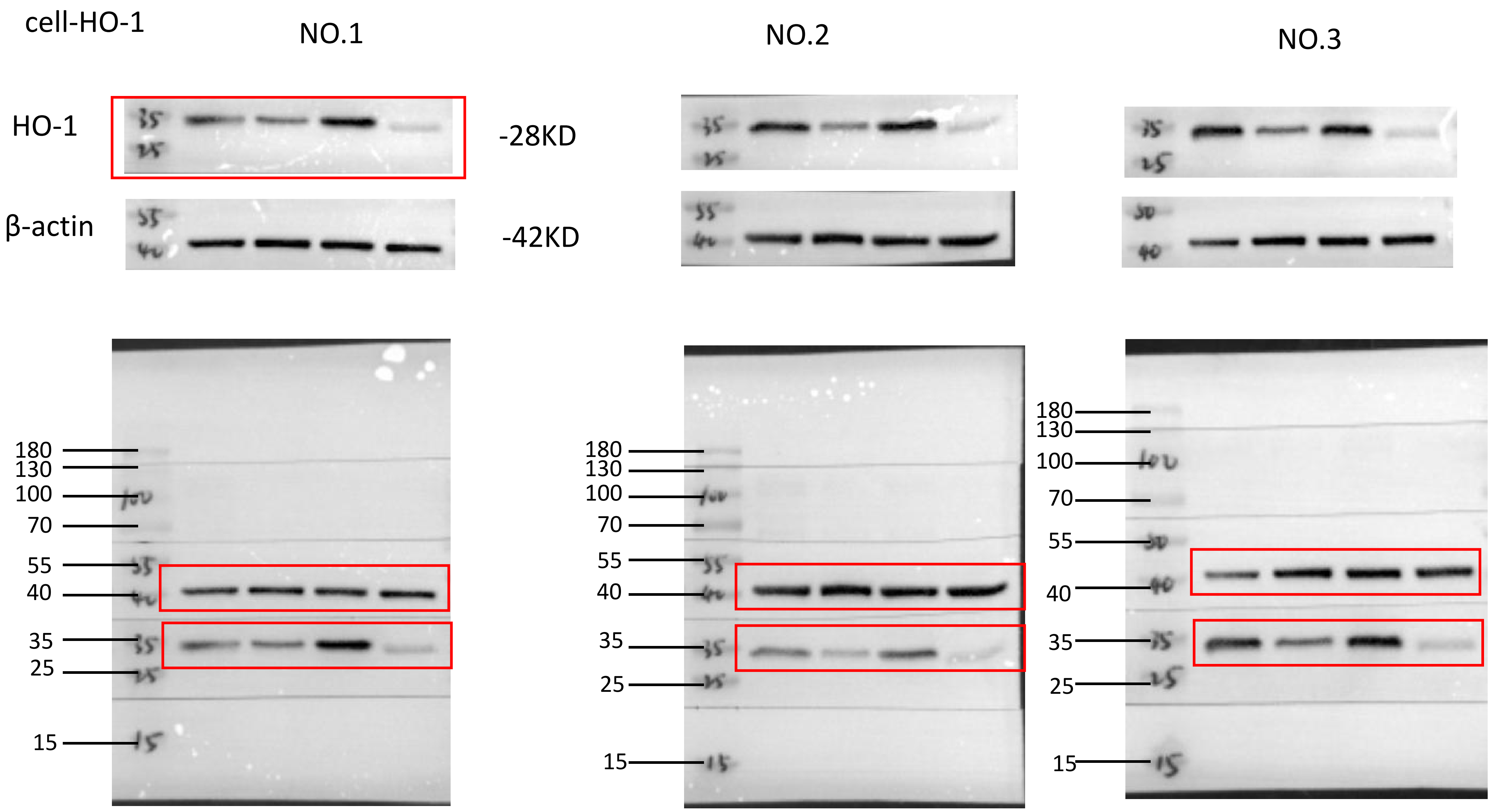

cell-HO-1
NO.1
NO.2
NO.3
HO-1
β-actin
-28KD
-42KD
180
130
100
70
55
40
35
25
15
